# Structural Defects and Processing Limitations for Polymer Film Blowing Applications: A Comprehensive Review of Conventional and Emerging Sustainable Technologies

**DOI:** 10.3390/polym17243314

**Published:** 2025-12-15

**Authors:** Ilke Pelgrims, Annabelle Verberckmoes, Ignatii Efimov, Paul H. M. Van Steenberge, Dagmar R. D’hooge, Mariya Edeleva

**Affiliations:** 1 Centre for Polymer and Material Technologies (CPMT), Department of Materials, Textiles and Chemical Engineering, Ghent University, Technologiepark 130, 9052 Ghent, Belgium; ilke.pelgrims@ugent.be (I.P.); annabelle.verberckmoes@ugent.be (A.V.); ignatii.efimov@ugent.be (I.E.); 2 Laboratory for Chemical Technology, Department of Materials, Textiles, and Chemical Engineering, Ghent University, Technologiepark 125, 9052 Ghent, Belgium; paul.vansteenberge@ugent.be; 3 Centre for Textile Science and Engineering, Department of Materials, Textiles, and Chemical Engineering, Ghent University, Technologiepark 70a, 9052 Ghent, Belgium

**Keywords:** film blowing, plastic defects, mechanical recycling, multilayer films, process control

## Abstract

This review provides an in-depth look at the key process limitations and (structural) defects encountered in the production of polymer films via film blowing extrusion technology. Film blowing is the most widely used method for producing plastic films across various industries, with its increasing demand driven by flexible packaging needs. Overcoming the challenges of this complex production process is essential for ensuring high quality and meeting the growing demand for modern applications, taking into account polymer circularity. In the first part of this paper, the focus is on conventional films, generally polyolefin single-layer films. Common defects such as bubble instability, gauge variations, wrinkles, melt fractures, optical defects, blocking, and surface imperfections like fish eyes are discussed. The most important causes behind these issues are elaborated on, including various molecular and processing parameters, with this paper also offering practical mitigating strategies. In the second part, the specific process limitations and defect types associated with emerging sustainable film technology are focused on, covering films made from recycled materials, biodegradable polymers, polymer blends, and multilayer and machine-direction oriented (MDO) films. While these innovative films offer significant advantages in terms of sustainability and property enhancement, they also present additional points of attention. Also, effective mitigation strategies for addressing these technical issues are incorporated. Overall, this study provides a comprehensive review of film blowing defects, contributing to improved process control, reduced waste, and the production of high-quality films that meet modern requirements. By identifying the root causes of common defects and discussing viable solutions, this review plays a key role in advancing the efficiency, consistency, and sustainability of film blowing technology by presenting a combined experimental and modelling approach that can be used in future work.

## 1. Introduction

Blown film extrusion is the primary method used to produce plastic films for, e.g., food, non-food packaging, agricultural, and construction sector [1,2,3]. Due to its versatility, it has seen tremendous growth in recent decades, making it one of the fastest-growing processes in plastic manufacturing [4]. In Europe alone, approximately 13 to 15 million tonnes of plastic films are produced annually, with the polyethylene (PE) type accounting for approximately 65% of this total [5]. Low-density PE (LDPE) and linear low-density PE (LLDPE) are the most widely used types of PE, though high-density PE (HDPE) is also commonly utilised [3,6,7]. Polypropylene (PP) is the second most widely used polymer type for flexible films, and is becoming increasingly popular in the production of flexible films [3]. In 2020, the total production of PE and PP for all applications in Europe reached approximately 25 million tonnes, with around 11 million tonnes used for the production of flexible films [3]. Together, PE and PP accounted for roughly 37% of Europe’s total plastics production in 2022 [8].

Globally, plastics production exceeded 400 million tonnes in 2022 [8]. Packaging remained the largest market for converted plastics, accounting for 39% of the total and underscoring the dominant role of flexible films [8]. For example, the LDPE market was 22.6 million tonnes in 2024 and is projected to reach 32.6 million tonnes by 2035, with film extrusion driving nearly 70% of demand [9]. The HDPE market stood at about 52 million tonnes in 2024 and is expected to grow to 70 million tonnes by 2035, largely due to packaging, which represents roughly 32% of HDPE use [10].

The demand for flexible films continues to grow steadily, driven by the increasing need for convenience products and versatile packaging. The COVID-19 pandemic also had a significant impact. While the overall plastic demand declined in 2020, due to the economic slowdown, the demand for flexible films increased, driven by a rise in home cooking, supermarket shopping, and food delivery, which led to a greater need for food packaging [3]. Current trends, such as energy-efficient machines and multilayer films, continue to drive the growth of flexible films [11]. The blown film market is expected to experience strong growth, driven by rising demand for plastic films in sectors such as packaging, agriculture, and construction. In 2022, the market was valued at USD 3.5 billion and is projected to reach USD 5.9 billion by 2030, with an estimated compound annual growth rate (CAGR) of 6.7% from 2024 to 2030 [12]. Another source estimates the global blown film extrusion lines market at USD 5.3 billion in 2023, expected to grow to USD 7.6 billion by 2028 (CAGR 7.4% during the forecast period) [13]. This expansion is primarily driven by the increasing demand for lightweight, flexible packaging and sustainable material solutions [12].

**Figure 1:**
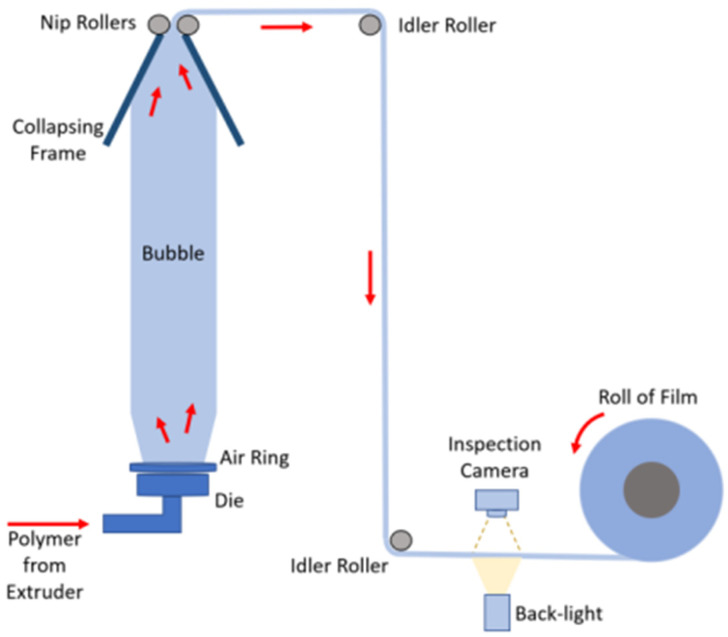
Film blowing process with an inspection camera for defect detection [6].

The above growth numbers underscore the importance of ensuring consistency and quality in production [4,14]. However, (structural) defects in blown films present significant challenges [15,16,17]. The blown film manufacturing process, in which molten plastic is extruded through a circular die, inflated into a bubble, and then cooled and flattened into a uniform film, is complex and requires precise process control [2,6]. Figure 1 illustrates this process, showing important components such as the die, air ring, and collapsing frame. Factors such as material purity, temperature, screw speed, cooling rate, and winding speed must be carefully controlled, as even minor variations can lead to defects [6,17,18,19]. Such defects can arise from various sources, with temperature fluctuations, improper tension, and impurities within the production line playing an important role [14].

While packaging films may seem straightforward to make, they must meet strict technical standards, with factors such as friction, thickness, sealability, and shrinkage determining the performance. Rising plastic costs further emphasise the need to minimise thickness while maintaining quality, making defect control even more critical [4]. As plastic materials can account for up to 70–90% of production costs, reducing waste and maintaining tight tolerances are essential for cost-effective production [14]. The quality of polymer films is typically assessed based on the mechanical properties, optical properties, and crystalline structure, although the required evaluation methods are often time-consuming and destructive. To improve efficiency, attribute-based defect detection methods, such as inspection cameras (as shown in Figure 1), are increasingly used to identify defects based on specific characteristics [6]. In large-scale industrial production, continuous inspection is essential for maintaining consistent quality while reducing reliance on costly and labour-intensive manual tests. Moreover, early defect detection allows for corrective adjustments to improve the film’s overall quality [6].

With growing sustainability concerns, there is also an increasing pressure to develop recyclable or natural/biodegradable flexible films [3,7]. These sustainable materials introduce additional processing difficulties, and they currently exhibit lower quality than conventional films. This results in delays, higher failure rates, and discourages the use of recycled or biodegradable materials. In the future, the use of sustainable materials will become essential due to stricter regulations aimed at reducing environmental impacts. For instance, the EU’s minimum recycling targets for 2030 will present new challenges, mainly due to the variability in material quality between batches. This can result in poor extrusion, low-quality films, and limited use of post-consumer recycled materials. Applications are also hindered by odours and impurities in the resin [20].

Recycling is critical for a circular economy and reducing plastic waste, yet only a small portion of flexible films are currently recycled. In 2020, 40% of flexible films in Europe were collected, but only 18% were actually recycled. This rate must increase, and solutions must be found to achieve this without compromising quality [3]. A key barrier to improving this rate is the logistical challenge of collecting post-consumer film waste. These films are typically thin and low-density, resulting in high-volume waste that is costly to transport, and they often contain contaminants such as mixed polymers, paper, organic residues, or soil [21,22]. Such contamination requires complex sorting and washing, and the films’ low density and high moisture can disrupt recycling, reducing mechanical efficiency [21].

Moreover, the European Union (EU) aims to ensure that all plastic packaging becomes recyclable, reusable, or compostable by 2030, making biodegradable materials particularly interesting [23]. These materials are designed to degrade more quickly in the environment, potentially reducing long-term plastic pollution. Additionally, they help reduce reliance on fossil resources. However, biodegradable films face their own specific processing sensitivities, as they are highly responsive to conditions such as temperature, which complicates their use. Considerable research is still needed to enhance the performance of films made from these materials.

A promising solution to improve the properties of biodegradable films and films, in general, is to blend the polymer resin with other polymers. By combining different polymers with their own specific properties, the overall material properties can be optimised. However, this creates additional difficulties, as the materials must be carefully combined to ensure compatibility. Preferably, they should have (very) similar physical, chemical, and rheological properties to ensure miscibility and processability under the same conditions. Moreover, the polymers must complement each other’s characteristics without negating their favourable properties.

Another approach to combining multiple materials to improve material properties is the multilayer approach (although the recyclability of such films could be less trivial). These films can offer a combination of properties like barrier protection (against water, oxygen, and ultraviolet (UV) light), strength, sealability, and flexibility [24]. They are relevant for specific performance requirements, such as in food packaging, pharmaceuticals, and industrial packaging. Multilayer films thus allow for greater customisation and optimisation of a wider range of properties. For these packaging materials, however, the situation is more complex, requiring stability in each layer and effective adhesion between them [22]. Another technique to improve mechanical, optical, and barrier properties is to stretch the films in one direction, known as machine direction-oriented (MDO) films [25,26]. This must be done with accuracy, under the appropriate processing conditions and using suitable materials, as these films are particularly susceptible to defects [27].

This review provides insights into the key issues and defects in blown film production, along with their causes and mitigation strategies, complementing previous reviews [4,28,29,30] that deal more with the general features of films and focus less on the defects as such. It covers both conventional films, typically consisting of (single-layer) polyolefins, and advanced films either made from innovative materials or using emerging technologies. This review aims to set the key elements that need to be investigated in view of reaching targets such as improved control, reduced waste, and the production of high-quality films, covering both conventional film production and the more recent sustainable film technologies. Minimising defects is crucial for economic sustainability and to meet the growing demand for plastic films. This study examines the common problems in blown film production, identifies the root causes of defects, and discusses possible solutions for different types of blown films. By focusing on these factors, process efficiency and quality will be increased, resulting in more defect-free films that match the wide range of current requirements.

Since Rosato’s 1998 work [14], which provided a broad overview of blown-film production, several studies have addressed specific aspects of blown-film behaviour, film defects, and process troubleshooting. However, a detailed, systematic, and up-to-date review that examines the underlying causes, mechanisms, and mitigation strategies of defects across both conventional films and emerging sustainable or advanced film types is still lacking. The present study brings together these scattered insights and integrates existing knowledge on new materials, processing techniques, and quality-control methods, offering a comprehensive and structured perspective on defects in film-blowing processes. By synthesising findings from past decades of research with recent developments in sustainable materials, this work aims to support improved process control, reduced waste, and higher film quality, while also outlining key directions for future research.

## 2. Conventional Commodity Films

### 2.1. Most Common Defects

In blown-film production, product-related issues include not only visible or structural defects (e.g., gauge variation, melt fracture, wrinkles) but also broader quality deviations such as reduced mechanical performance, optical deterioration, and processing instabilities. In this section, both defect-related and non-defect quality issues are addressed, together with their causes and mitigation strategies.

Film blowing is a complex and delicate process with multiple technical considerations, which demand careful attention at every step to ensure a smooth production. It is one of the most sensitive polymer processing technologies, because it is highly susceptible to changes in conditions [31]. One of the primary technical issues lies in optimising machine settings, as balancing the speed, temperature, and pressure profile of the extruder is essential for a steady output [18,32]. Operators must be proficient in achieving the required bubble geometry, understanding the impact of each process variable on the bubble’s characteristics, and being able to control multiple factors simultaneously [33]. Consistent conditions throughout the process are thus crucial for maintaining uniform film thickness and quality. Even minor changes in these parameters can affect the final product, requiring continuous monitoring and adjustments to maintain the desired properties [32]. Specifically, controlling the cooling process after the film is blown is essential for good quality, as well as correctly managing the tension of the film during winding to ensure smooth, consistent rolls.

The use of different plastic materials, each with unique flow characteristics, adds complexity and requires precise process adjustments for each material [4]. Additionally, optimising energy consumption while maintaining production speed is another processing difficulty [32]. Even minor missteps in managing these different factors can have a significant impact. The interplay of all these parameters demands continuous attention to ensure optimal results [18]. If these factors are not carefully controlled, they can disrupt production, possibly resulting in different defects.

**Figure 2:**
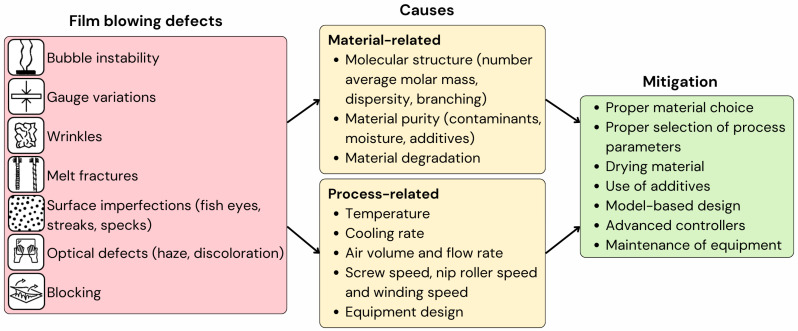
Overview of the most common film blowing defects, along with their main causes and mitigation strategies.

The defects that occur most often in blown films are bubble instability, melt fracture, gauge variation, optical defects, blocking, and surface defects like fish eyes, specs, and wrinkles. Their main causes, due to significant changes in molecular-scale characteristics or processing parameters, as well as several proposed mitigation strategies, are summarised in Figure 2. A comprehensive breakdown of these common defects, including their specific causes and mitigation strategies, can be found in Table 1. In what follows, a more detailed discussion is included.

**Figure 3:**
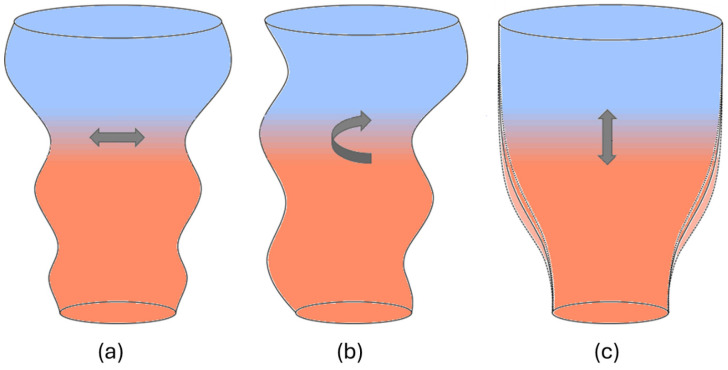
Film blowing defect of bubble instability: different types (**a**) draw resonance (horizontal arrows show thickness oscillation), (**b**) helical instability (curved arrow shows twisting motion), and (**c**) frost line height instability (vertical arrow shows frost-line fluctuation). Blue represents the cooled region and red the hotter molten film.

Bubble instability refers to irregularities in the shape, size, and position of the air bubble, which can lead to disruptions [34,35,36]. Maintaining a stable bubble is crucial for uniform film production because instability leads to non-uniform film thickness and layflat width, affecting the film’s properties. The three primary types of instabilities observed are draw resonance, helical instability, and frost line height (FLH) instability, as illustrated in Figure 3 [37]. Draw resonance occurs if periodic oscillations in the bubble’s diameter cause variations in film thickness [34,35]. Helical instability involves the bubble moving around its axis, while FLH instability results in fluctuations in the position of the frost line (the transition from molten to solid film) [34,37]. These instabilities are typically caused by several factors, including very high or very low blow-up ratio (BUR) (the optimal BUR for stability typically ranges between about 2.0 and 3.5), low take-up ratio (TUR) (below 15 to 20), excessive melt temperature relative to the resin recommendation, high cooling rate or uneven airflow, and poor polymer properties, due to air drag forces or local thinning of the film [36,37]. By controlling these parameters, bubble stability can be improved, and film properties can be optimised [34,36,37]. The material’s molecular structure, and thus the type of catalyst and comonomer content in, e.g., polyolefin film production, also plays an important role. For example, LDPE, with its strain-hardening behaviour in elongational flow, tends to be more stable, while materials with narrower molecular weight distributions (MWD), like HDPE and PP, are generally less stable [36,37,38].

**Figure 4:**
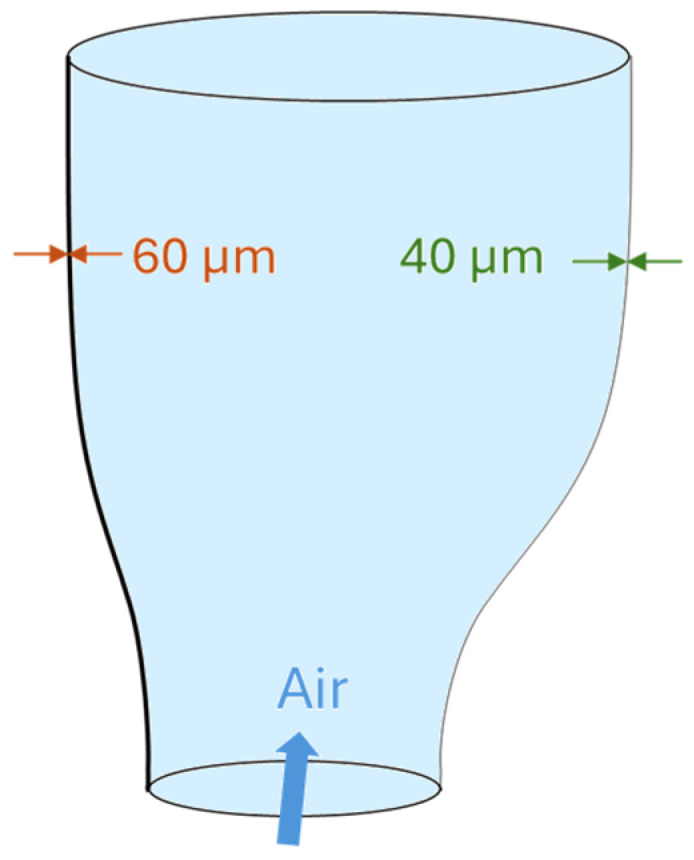
Gauge variation in the film caused by improperly oriented airflow during film blowing.

Gauge variation refers to inconsistencies in film thickness across the film width, which can negatively impact the final product’s quality [39,40]. This issue is strongly connected to the interaction of several air stream parameters, including air velocity, air temperature, air flow direction (as illustrated in Figure 4), and air volume, which directly impacts the uniformity of the melt stretching [39]. Equipment issues, such as misaligned or dirty (e.g., due to degraded material buildup) dies and air rings, can restrict material flow and affect cooling [31,37,41]. Moreover, variations in the melt quality, such as temperature variations exceeding a few degrees relative to setpoint, pressure, viscosity, or resin feed rates, can disrupt uniform polymer flow and also contribute to thickness variations [37]. Gauge variation can occur in both the machine direction (MD) and transverse direction (TD). In the MD, variations are often linked to extruder problems or inconsistent line speeds, with typical fluctuations becoming noticeable when the MD thickness variation exceeds about 5 to 10%. In the TD, issues with die alignment or uneven air distribution are common causes [31]. To reduce gauge variation, it is essential to optimise process parameters, ensure proper equipment alignment, maintain consistent airflow, and utilise advanced technologies like capacitive sensors for better thickness control [14,31].

Wrinkles are a common quality issue in film extrusion, characterised by irregularities in the film such as creases, peaks, and troughs across its width, leading to significant waste [14,42]. They are caused by several factors during the extrusion and winding processes, and are generally attributed to uneven stretching or improper equipment alignment [31]. They can result from equipment issues like misaligned rolls or collapsing frames, large gaps between collapsing frames, uneven die settings, and excessive resistance, as well as processing factors like tension, temperature, and line speed. Film density, thickness, and uneven material distribution also contribute to wrinkle formation [42]. Using higher-density plastics (e.g., HDPE) increases stiffness but also makes the film more susceptible to wrinkles, as their reduced stretchability causes uneven tension during processing [14]. Additionally, nonuniform cooling or a high frost line, in particular more than 8 to 9 die diameters, can create uneven molecular orientation, leading to differential shrinkage and warping, causing wrinkles [37,43]. Wrinkles can appear in two directions, with MD wrinkles from compressive forces or misaligned rolls, and TD wrinkles from sagging or poor winding control [44]. To prevent wrinkles, it is crucial to ensure a proper equipment setup, uniform tension, and consistent cooling during the film blowing process [31,37].

Melt fracture, also called sharkskin in extreme cases of deformation, is a surface defect caused by high shear stresses during extrusion, leading to a high roughness or washboard-like textures [44,45]. Polymers usually flow in continuous streamlines, but melt fracture occurs when this flow ruptures. It takes place in case the critical shear rate, as determined by the polymer type and grade, die geometry, and temperature, is exceeded [45,46]. Polymer processing additives (PPAs) can, however, be incorporated to increase the shear rate at which a polymer can be processed, in order to widen its processing window and improve flow stability [45,46,47]. Melt fracture typically occurs at the die lip. It can be reduced by increasing the temperature, lowering the resin viscosity (lower average molar mass or molecular weight (MW) or broader MWD), polishing the die lips, using low-friction coatings, widening the die gap, or adding PPAs [37,44].

Surface defects like lines, streaks, and foreign specks are often caused by degraded resin, foreign material, or die issues [14,37]. More specifically, black dots typically result from degraded resin, due to high temperatures, stagnant areas, or material left in the die during startup or shutdown [37]. Contamination may arise from excessive scrap or regrind polymer or foreign materials in the melt [14]. Lines and streaks can result from die lip imperfections, scratches from take-down equipment, or welding lines. Poor mixing or improper die alignment can also cause streaking [37]. Corrective actions include cleaning the die, repairing die lips, removing degraded resin, checking materials, limiting scrap use, and adjusting temperatures. Further measures may involve replacing the screen pack, adjusting die bolts, improving melt homogenisation, switching to a spiral die, or changing filters to reduce contamination and enhance quality [14,37].

**Figure 5:**
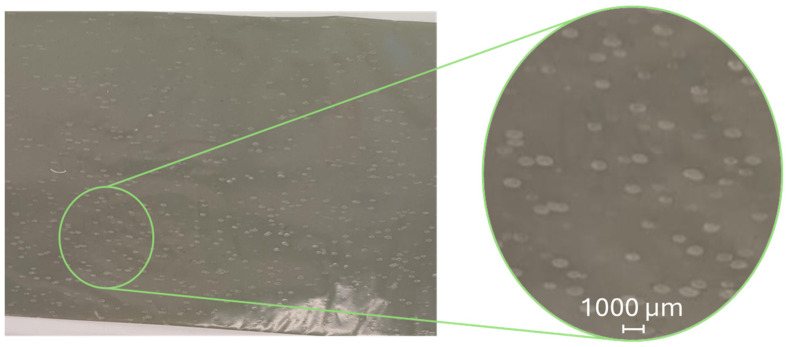
Blown film of recycled LDPE with visible fish-eye defects, with the scale bar indicating the approximate defect size.

Another common surface defect in blown film is the issue of fish eyes, also called “crystal points”. They are micro-entities (typically ranging from a few tens to a few hundred micrometres in size) in plastic films, typically caused by non-intentionally added substances (NIAS) [48]. They are especially common in recycled films, with Figure 5 showing an example. These defects appear as small dots or irregularities, ranging from tens to hundreds of micrometres, affecting the film’s optical transparency, appearance, and mechanical properties [49]. The plastic forming fish eyes differ from the primary material, with a wider MWD, lower crystallinity, and more branching. Films with fish eyes typically contain more volatiles than the primary plastic but share a similar crystallisation temperature, indicating they are composed of the same type of polymer [48]. They usually occur during the extrusion process, specifically during film pulling [17]. They result from imperfect mixing, contamination, process instability, or residues left after maintenance [48]. Reducing fish eyes requires optimising process conditions, adjusting screw speed, using finer sieves, adjusting additives, and ensuring proper melt homogenisation [14,48].

**Figure 6:**
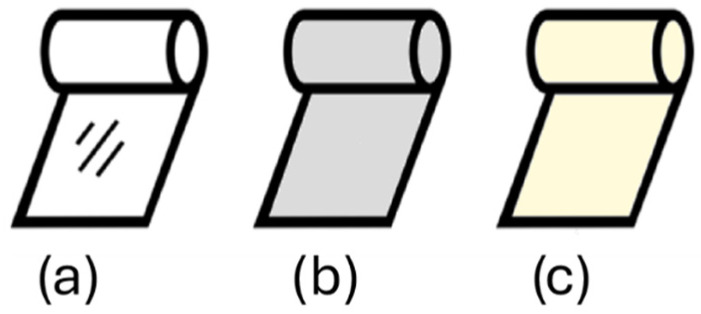
Conceptual examples of (**a**) a clear, transparent film; (**b**) a hazy film; and (**c**) a film with discolouration.

Optical defects, such as haze (Figure 6b), mainly arise from surface roughness, which is caused by extrusion defects and crystallisation defects [50,51,52,53]. Extrusion defects result from an uneven elastic response of the molten polymer once it leaves the die, while crystallisation defects arise from crystal formation on the surface [50,51]. Major causes include melt elasticity, where higher elasticity leads to more defects; less controlled crystallization kinetics, in which the crystallization rate and cooling affect surface roughness; molecular structure, where large molecules and long chain branches increase melt elasticity; and processing conditions, such as TUR, BUR, and FLH, which affect the cooling rate [51,52,53]. In commercial films, haze values above 5–10% (ASTM D1003 [54]) become visibly noticeable, although acceptable levels depend on the application [55].

Measures to reduce haze include mechanical treatment to reduce melt elasticity, modification of polymer composition by using polymers with a lower melt elasticity, process optimisation to adjust processing conditions, and multilayer films, for which a thin, low-haze outer layer improves the overall optical properties [51,52,53]. Despite this, internal scattering plays a smaller role than surface roughness, and it can also contribute to haze [51,53].

Discolouration (Figure 6c), another form of optical defect, is a type of degradation that occurs due to various factors, often involving chemical reactions within the polymer matrix [56,57,58]. In polyolefin films, discolouration can result from the breakdown of antioxidants, which can be triggered by factors such as high temperatures, exposure to nitrogen oxides, ionisation, or insufficient stabilisers. The presence of certain pigments can also cause colour changes through overoxidation [58]. For protein-based films, lipid oxidation or browning reactions between amino acids and carbonyl groups can lead to discolouration [57]. To prevent discolouration, using stabilisers and antioxidants and maintaining proper production and storage conditions is essential [57,58].

**Figure 7:**
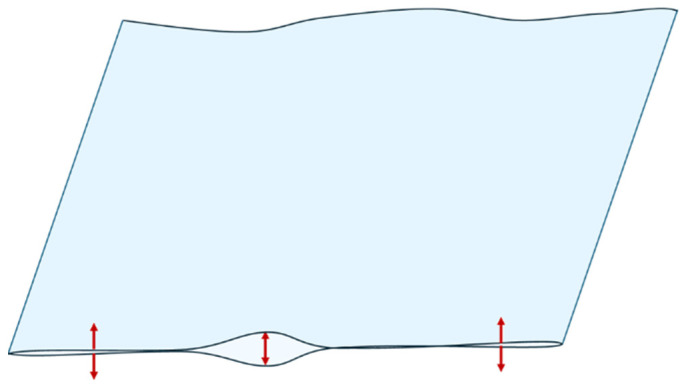
Blocking in a double-folded film, caused by sticking of the inner surfaces.

Finally, the issue of blocking refers to the unwanted sticking together of film layers, which complicates the film opening and further processing [4,59,60,61]. This occurs due to intermolecular forces between the surfaces of the film [59]. Figure 7 illustrates the blocking effect in a double-folded blown film, in which the inner layers adhere to each other during or after winding, making separation difficult. Various factors contribute to blocking, such as molecular properties of the materials, including density, MW, MWD, melt elasticity, and crystallinity [4,59]. Additionally, process conditions like temperature, cooling, mould opening, and BUR play significant roles. The use of additives such as antiblock and slip agents can help reduce blocking [61]. A rougher surface morphology of the film, achieved through material selection or additives, reduces the contact area, thereby reducing blocking [59]. The effectiveness of antiblock agents and PPAs is interdependent, and their performance can be influenced by how they are combined, with a combined masterbatch sometimes giving a worse antiblocking efficiency [59,60].

**Table 1:** Causes and mitigation strategies of common film blowing defects *.

Defect	Causes	Mitigation Strategies
**Bubble instability**	Very high/low BUR	Decrease/increase air volume; decrease/increase nip roller speed [34,36,37]
	Low TUR	Increase winding speed; reduce cooling rate [34,36]
	High melt temperature	Lower extrusion temperature; reduce screw speed [34,36,37]
	Excessive/uneven cooling	Balance airflow; clean air ring; optimize cooling temperature; use of double air rings/both internal and external bubble cooling [34,36,37]
	Undesirable molecular properties	Use materials with higher MW(D) or more branched structure; add stabilizers [36,47]
	Contaminants (NIAS)	Ensure polymer purity (e.g., filtration); remove any contamination from die [37]
	Nonuniform die	Verify die gap uniformity [37]
**Gauge variation**	Excessive, insufficient, or uneven air flow	Adjust cooling air rings for uniform flow; balance airflow; optimize air velocity [39]
	Misaligned/dirty die or air rings	Clean and align dies and air rings [31,37]
	Melt quality variations	Stabilize barrel temperature, gravimetric feeders/constant level in hopper; clean filters; dry materials; uniform resin batches [31,37]
	Line/draw speed variations	Synchronize line speed and tension (rollers) control; lower tension on nip rollers [31]
**Wrinkles**	Misaligned rolls or collapsing frames	Align collapsing frames and rollers; increase span between rollers; decrease friction on rollers [37,42]
	Non-uniform or excessive tension	Optimize tension control and film flattening; adjust winding torque, then nip pressure, then finally web tension (match rotation speed to draw speed) [31,42]
	Non-uniform or inadequate cooling	Balance airflow; clean air ring; adjust air ring for uniform flow [31,37]
	Poor winding control	Improve winding equipment setup [42]
	Uneven material distribution	Improve mixing (in die); higher purity polymer; filter impurities [42]
	Low-density polymer	Use polymers with higher density; blend with other polymers [14,42]
**Melt fracture**	High friction in die	Lower resin viscosity (lower MW or higher MWD); polish die lips; using low-friction coatings; add PPAs/fillers [37,44,46,47]
	Low melt temperature	Increase extrusion and die temperature [37,44]
	Die gap too narrow	Widen die gap [37]
	Undesirable molecular properties	Use polymer with lower MW, higher MWD, and higher degree of branches [44]
**Fish eyes**	Poor mixing	Proper melt homogenization; use proper screen pack [14,48]
	Contamination (NIAS)	Use finer sieves; use higher purity resins (e.g., virgin); remove residue after maintenance; clean screw, barrel, and die [14,48]
	Process instabilities	Optimise process conditions (e.g., increasing die temperature and screw speed) [48]
**Streaks, specks**	High temperatures	Lower temperatures [14,37]
	Stagnant areas	Remove degraded resin [37]
	Material left in die during startup or shutdown	Clean/check die and barrel [14,37]
	Excessive scrap, regrind polymers or NIAS	Check materials (e.g., purity); limit use of scrap; replace screen pack; use better/change filters [14,37]
	Die lip imperfections, scratches from take-down equipment, or welding lines	Repair die lips; adjust die bolts; increase melt temperature [14,37]
	Poor mixing	Improve melt homogenization; better mixing [37]
	Improper die alignment	Switching to spiral die; realign die [14,37]
**Haze**	High melt elasticity	Mechanical treatment [51]
	Undesirable molecular structure (high crystallisation rate)	Change molecular composition (lower MD, narrow MWD, linear or SCB polymers) [51,52]
	Low BUR	Increase air volume in bubble [14]
	Inadequate cooling	Increase cooling rate [19,62]
	Presence of moisture	Drying the resin [63]
	Addition of additives	Use of masterbatches, addition of interfacial agents (such as PEG and PCL), mechanical treatment [45]
	High screw speed	Decrease screw speed [64]
	Low extrusion temperature	Increase extrusion temperature [14]
	Impurities	Add compatibilizers, improve mixing, filtration, maintenance extruder [31]
**Discoloration**	High temperatures	Decrease extrusion temperature [57,59]
	Exposure to nitrogen oxides	Store films in controlled environments; use NO_x_-resistant materials or additives [57,58]
	Ionization	Use UV stabilizers or protective coatings; limit exposure to UV light [58]
	Presence of certain pigments	Use stable (under heat and light) pigments [57,58]
	Time	Use UV inhibitors (stabilisers) and antioxidants; improve packaging and storage [57,58]
	Moisture	Dry material; control humidity in processing environment; add moisture-absorbing agents; use moisture barriers during storage [57]
**Blocking**	Inadequate cooling	Increase cooling rate [61]
	High temperature	Decrease extrusion temperature [5]
	Undesirable molecular properties	Use polymers with higher MW(D) and better branching [4,59]
	Low melt elasticity	Use polymers higher melt elasticity (low MW, narrow MWD, and low level of branching); add anti-block agents (e.g., slip additives) [59,60,61]
	Low crystallization rate	Increase cooling rate; use polymers higher crystallinity [49,50]

* Note that qualitative terms (e.g., low, high, slow) are used throughout this review to indicate relative trends. Their absolute values cannot be defined universally, as they depend strongly on the specific film-blowing equipment, polymer grade, formulation, processing settings, and ambient conditions. These relative terms are included only to indicate the direction of change relevant for troubleshooting.

### 2.2. Process-Related Causes of Defects

In blown film production, achieving high-quality products heavily relies on carefully controlled processing parameters. Even small deviations in these parameters can cause a variety of defects that affect both the visual appeal and functionality of the film [65,66]. Poorly chosen settings can lead to numerous defects, affecting the film’s appearance, mechanical properties, and overall performance [14].

**Figure 8:**
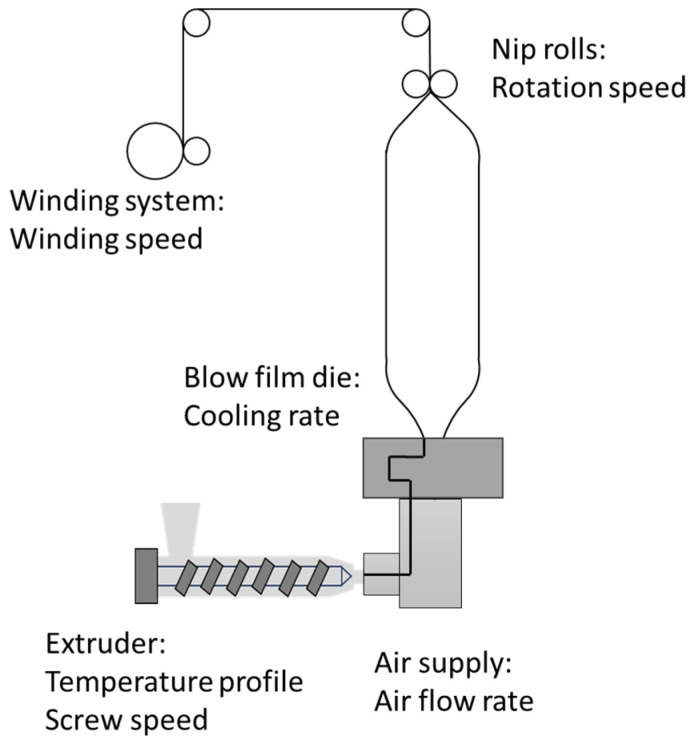
Schematic overview of the film blowing process, with the most important processing parameters indicated.

Figure 8 provides an overview of the most relevant processing parameters that can be actively adjusted during production. These include screw speed, temperature profile, cooling rate, air flow rate, nip roller speed, and winding speed. Their influence on defect formation will be discussed in detail in the following parts. Processing parameters like BUR, TUR, and output rate, which are often discussed in the literature, are not directly discussed here because these parameters depend on various other processing settings and cannot be adjusted independently during the film production process. BUR is mostly controlled by how much air is injected into the bubble, but other factors like melt temperature and cooling air flow also influence it. TUR, for example, is influenced by parameters such as nip roller speed and temperature, while the output rate is indirectly controlled through factors like screw speed and temperature.

#### 2.2.1. Temperature

Among the most critical factors is temperature control. The extrusion temperature (profile) plays a central role in determining film clarity, strength, and bubble stability [4,14,67]. Typically, the extrusion temperature is selected above the melting temperature T_m_ for semi-crystalline polymers and significantly above the glass transition temperature (T_g_) for amorphous ones. In practical terms, it means that +20–50 °C above T_m_ for most semicrystalline thermoplastics (or ~5–15%) is the typical engineering range for the optimal temperature selection. For amorphous polymers which have no T_m_, the selection of the processing temperature relies on the T_g_ value, with T_g_ + 80–150 °C or ~30–70% above T_g_ being typically selected [68,69,70].

Extrusion temperatures for film blowing are selected relative to a polymer’s thermal transitions because melt rheology depends sensitively on chain mobility. For semicrystalline polymers, processing must occur above the melting temperature (T_m_) to ensure full crystal dissolution and achieve a workable viscosity; small temperature increases above T_m_ significantly reduce viscosity through Arrhenius-type behaviour while excessive overheating lowers melt strength and destabilizes the bubble [68,69]. Amorphous polymers require temperatures well above the T_g_ because chain relaxation times remain extremely long near T_g_, and practical flow occurs only at T_g_ + 80–150 °C [68]. The optimal window is therefore determined by balancing viscosity reduction, melt strength retention, and thermal-degradation limits, supported by rheological measurements that define the temperature at which the melt reaches the extensional viscosity necessary for stable film inflation [71,72].

If the extrusion temperature is set below the polymer’s processing window, e.g., significantly below the above-mentioned ranges, the polymer may not fully melt, leading to poor strength and a hazy appearance. Furthermore, low melt temperatures promote film haze due to an increase in crystallite growth [73]. Inadequate temperatures will also result in poor melt flow, uneven thickness, and surface imperfections like streaks or gel-like bubbles [14,19]. Insufficient heat may also prevent proper integration of additives, creating a non-uniform material that lacks the desired performance characteristics [74].

Higher extrusion temperatures, i.e., above T_m_, reduce the polymer melt viscosity, allowing for faster extrusion and increased output rate, boosting production efficiency. Moreover, higher melt temperatures lead to more rapid stress relaxation, higher orientation, and a higher impact strength [73]. However, overheating during extrusion (temperatures exceeding the polymer’s thermal stability limit, T_degradation onset_ as determined by TGA) can lead to polymer breakdown due to degradation, which impacts the film’s mechanical properties and appearance. As the polymer degrades, the film may exhibit signs such as discolouration, brittleness, and a loss of tensile strength. In some cases, an unpleasant odour may also develop, which is a clear indicator of polymer degradation [75]. Moreover, high temperatures can also destabilise the bubble, possibly causing variations in film thickness and asymmetry, and increasing the risk of film sticking (blocking) during winding [5,65,76]. Additionally, improper temperature control at the die lips may lead to melt fracture, a defect characterised by a rough surface or fine surface irregularities on the film [14,77]. This roughness can be mitigated by increasing the die temperature to facilitate a smoother melt flow [77]. The criteria for excessively high extrusion temperature are often determined as Tm + 60–80 °C for semi-crystalline and T_g_ + 150–200 °C for amorphous polymers, respectively.

**Figure 9:**
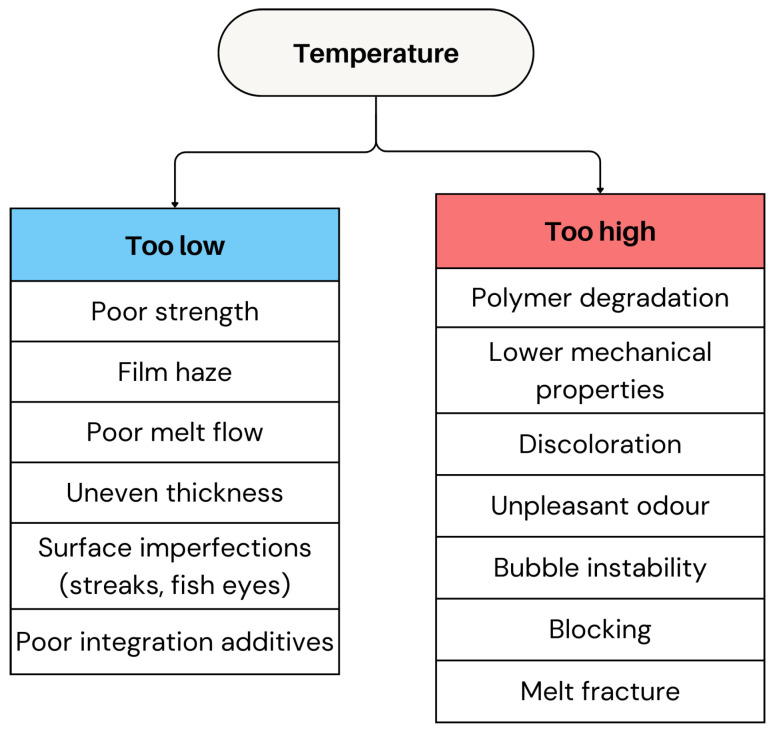
Overview of defects caused by improper temperature profiles (out-of-spec conditions *). * In this context, “out-of-spec conditions” refers to processing conditions that fall outside the recommended operating window for a given polymer and film-blowing setup. Qualitative terms (e.g., low, high, slow) are used throughout this review to indicate relative trends. Their absolute values cannot be defined universally, as they depend strongly on the specific film-blowing equipment, polymer grade, formulation, processing settings, and ambient conditions. These relative terms are included only to indicate the direction of change relevant for troubleshooting.

An overview of the various defects and processing challenges caused by both excessively low and high extrusion temperatures is presented in Figure 9. Maintaining precise temperature control during extrusion is essential to ensure that the polymer viscosity remains within spec. Variations in viscosity can compromise material properties and may place excessive strain on the screw and extruder drive, potentially leading to operational issues and product defects. To reduce defects caused by temperature fluctuations in the film blowing process, proportional temperature control devices can be used. By automatically adjusting heating across different zones, these devices help to maintain a stable polymer flow and minimise stress on equipment, which improves film quality [19].

#### 2.2.2. Cooling Rate

Cooling rate in film blowing is a critical parameter because it governs the solidification point, crystallization kinetics, and final molecular orientation of the film. Optimal cooling must strike a balance between rapid quenching, which preserves orientation and reduces crystallinity, and adequate cooling time, which stabilizes the bubble and prevents shrinkage or warpage. For semicrystalline polymers, cooling is directly linked to crystallization temperature (T_c_) and crystallization half-time: cooling should reduce the melt to below T_c_ quickly enough to freeze the bubble geometry, but not so rapidly that it suppresses desirable crystalline morphology or induces internal stress [78,79]. Amorphous polymers, lacking crystallization, solidify when the temperature approaches T_g_, and optimal cooling ensures sufficient relaxation before vitrification to avoid brittleness. Rheological and calorimetric measurements (DSC crystallization curves, T_c_ shifts, relaxation times) help determine the proper cooling rate, with air-ring design, throughput, and blow-up ratio adjusted to maintain bubble stability while achieving the targeted structure and optical properties [69].

The cooling rate has a direct impact on the FLH and polymer crystallinity, influencing both optical and mechanical properties [19,80]. A fast cooling generally results in a higher degree of TD orientation, improving the film strength in this direction [62]. Moreover, an increased cooling rate can improve stability [5]. However, beyond a certain threshold (if the cooling is too rapid relative to the polymer and ambient conditions), excessive cooling can reduce bubble stability, leading to uneven crystallisation, uneven film structures, internal stresses, and MD shrinkage [4,5]. This can result in poor clarity and poor mechanical properties [19,62].

**Figure 10:**
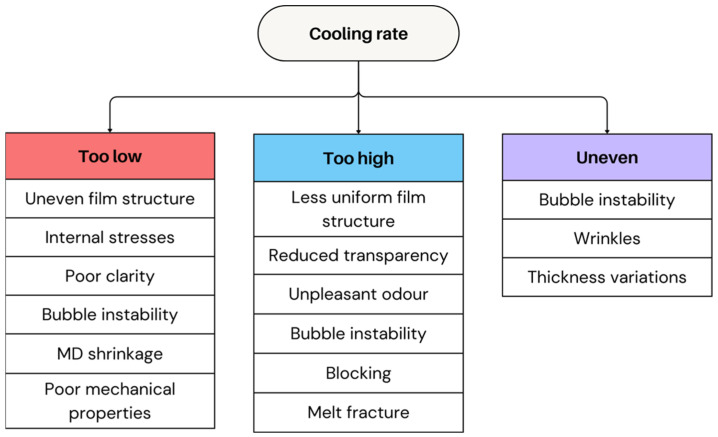
Overview of defects caused by improper cooling rate (out-of-spec conditions).

On the other hand, slow cooling allows more bubble expansion but may result in a less uniform film structure [4]. It also enhances crystallinity, which results in a stiffer product, although this can reduce transparency because the FLH increases [73]. Moreover, such cooling is more prone to blocking, which not only complicates unwinding but also lowers the film quality by degradation [4,61]. Uneven cooling across the bubble can also contribute to bubble instability, creating wrinkles and thickness variations [14,65,76]. Typical issues resulting from improper or uneven cooling rates are summarised in Figure 10. Optimising the cooling air distribution can help maintain bubble stability and reduce these defects.

#### 2.2.3. Air Volume and Flow Rate

The air flow rate (speed of air introduction) and air volume (amount of air in the bubble), closely connected to the BUR, are essential in determining the film strength, flexibility, and clarity. Air volume and flow rate in film blowing are selected to balance heat-transfer requirements, crystallization kinetics, and bubble stability. It is known that cooling capacity depends on the convective heat flux:

$$q=hA(T_{melt}-T_{air})$$

where the heat-transfer coefficient *h* increases with air velocity but plateaus due to boundary-layer limits. Adequate airflow must remove heat rapidly enough to reach the T_c_ or T_g_ without inducing bubble flutter or draw resonance. Optimal settings place the frost line at 1–3 die diameters and match cooling rate to melt strength and extensional rheology constraints [69,81].

A high air volume stretches the film in the TD, which can reduce tensile strength along the MD. This weakens the film’s resistance to tearing in specific directions, making it unsuitable for applications requiring uniform strength [64]. Moreover, it may lead to a less glossy surface appearance [4]. However, it enhances TD orientation and, consequently, the dart drop impact strength. Furthermore, a higher air volume inside the bubble improves strain at break and lowers elastic modulus [5]. A higher air volume improves the stability of the film [5]. Yet, if the BUR is too high (for instance, higher than 4), excessive pressure on the bubble can cause instability, leading to an irregular shape, especially if it contacts the die parts. In some cases, the bubble may even lead to rupture.

**Figure 11:**
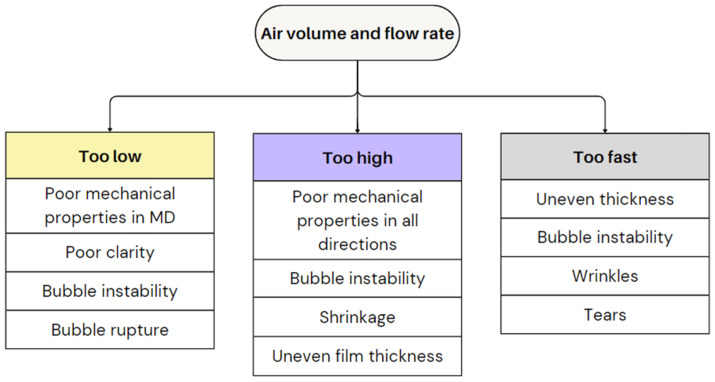
Overview of defects caused by improper air volume (out-of-spec conditions).

A low air volume, such as a BUR smaller than 1.5, in contrast, produces a thicker film with little orientation, leading to poor tensile strength, flexibility, and durability [64]. Additionally, insufficient air volume can cause bubble instability and shrinkage, resulting in an uneven film thickness [4]. The air volume or BUR should be matched to the winding speed to achieve balanced orientation in the film. Moreover, it is best to feed the air slowly to develop the bubble. If air is introduced too quickly or unevenly, it can lead to irregularities in the film thickness, instability in the bubble, or even defects like wrinkles or tears. Figure 11 illustrates the effects of suboptimal air volume and flow rate on bubble stability and film quality.

#### 2.2.4. Screw Speed

A proper screw speed is also key to quality, as incorrect settings can cause various film defects [18].

Screw speed in film blowing is selected to balance output, melt temperature, and bubble stability. Increasing screw speed raises shear heating and throughput, but excessive RPM reduces residence time, increases melt-temperature overshoot, and lowers melt strength—leading to draw resonance and bubble instability. Optimal speeds achieve the target output while maintaining a stable melt temperature profile predicted by energy balance:

$$\Delta T\propto \frac{\tau \dot{\gamma }}{\rho c_{p}},$$

where shear stress τ and shear rate $\dot{\gamma }$ increase with RPM [69]. Practical guidelines keep screw speed at 40–70% of maximum extruder screw speed to maintain homogeneous melting, adequate mixing, and consistent pressure [82].

A larger screw speed increases the material processing rate, thereby elevating the film’s output rate. However, excessive throughput disrupts flow stability, exacerbating surface defects like die lines [83]. An increased upstream melt flow amplifies extrudate swell and flow disturbances near the die lips, making die lines more pronounced, particularly in polymers like LLDPE and HDPE [84]. Setting the screw speed too high raises the melt temperature, which can make the film too fluid and difficult to control, leading to uneven thickness, bubble instability, and a loss of clarity [64].

**Figure 12:**
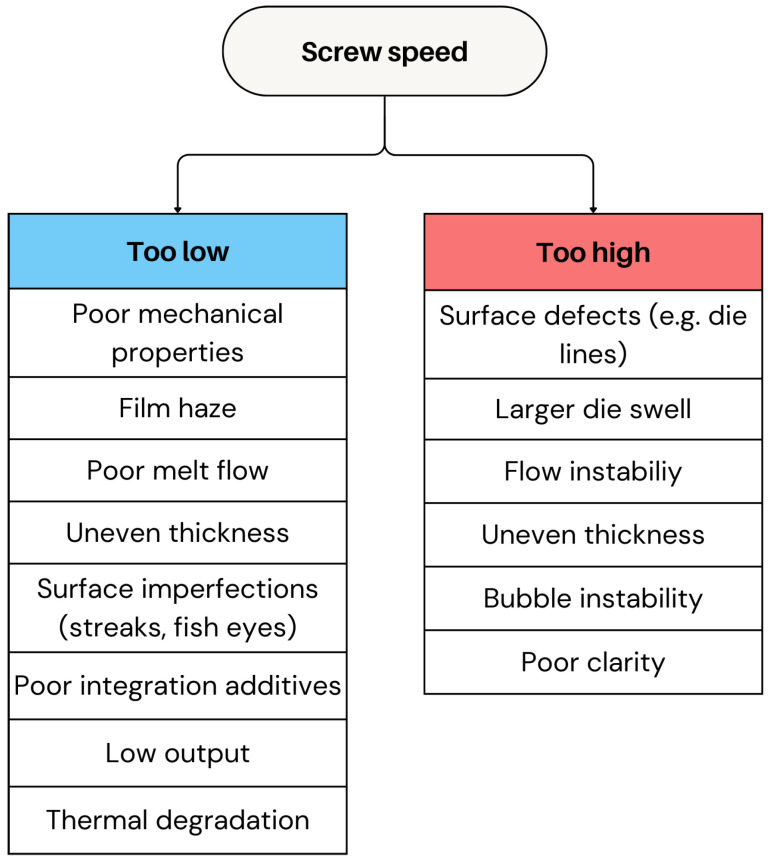
Overview of defects caused by improper screw speed (out-of-spec conditions).

Conversely, a low screw speed can result in poor melting because the polymer does not receive enough heat and shear to fully melt [85]. This affects the film’s structure and appearance (see Section 2.2.1). Moreover, a low screw speed increases the residence time (RT) of the polymer in the barrel, which can lead to thermal degradation as the material could be exposed to heat for too long. Additionally, it leads to a low output, reducing both production efficiency and turnover. Figure 12 shows the impact of both low and high screw speeds on the film performance.

#### 2.2.5. Nip Roller Speed

Nip-roller speed controls the take-up rate of the blown film, directly affecting the draw ratio, bubble stability, and final film thickness. The draw ratio, defined as $\mathrm{DR}=V_{\mathrm{nip}}/V_{\mathrm{die}}$, governs molecular orientation and uniformity. Optimal nip speed balances melt rheology and extensional viscosity: a low-viscosity or low-melt-strength polymer can overstretch, causing neck-in or flutter, while high melt strength resists excessive thinning [69]. Extensional viscosity, $\eta_{e}$, determines resistance to axial stretching under nip pulling, ensuring stable bubble inflation and uniform gauge [81]. Fine-tuning is guided by frost line height, melt temperature, and observed bubble behaviour.

**Figure 13:**
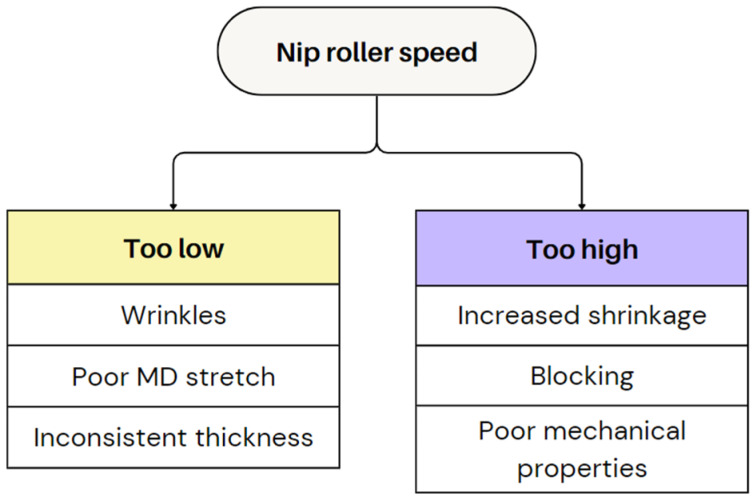
Overview of defects caused by improper nip roller speed (out-of-spec conditions).

A high nip roller speed stretches the film, making it thinner but excessive speed can over-orient the molecules, leading to increased shrinkage, blocking, and a reduction in mechanical properties [62]. A low nip roller speed produces thicker films but may cause wrinkles, poor MD stretch, and inconsistent thickness [14]. Figure 13 provides an overview of defects caused by an improper nip roller speed.

It is important to balance both nip roller speed, winding, and screw speeds. An adjustment in one of these parameters often impacts the bubble geometry and film dimensions. For example, increasing the nip roller speed without balancing the screw speed can unintentionally increase FLH and lay flat width, which complicates the process. Modern extrusion lines often feature automated systems that balance these speeds to maintain film quality and reduce the likelihood of defects [14].

#### 2.2.6. Winding Speed

Proper tension control during the winding process is essential for achieving uniform film rolls and preventing defects such as loose or deformed edges [14]. Winding speed is critical for maintaining film tension, preventing wrinkles, and controlling final mechanical properties. It is typically set slightly below, i.e., 3–5% lower, the nip-roller speed to maintain positive tension without overstretching. Optimal speed ensures uniform draw and stress distribution along the web, accounting for polymer viscoelasticity, residual stresses from cooling, and orientation frozen at the frost line [68,82]. Fine adjustments are guided by online tension monitoring and film property evaluation.

In general, a lower winding speed may allow for more uniform cooling and better film quality but can reduce throughput [62]. However, in case the winding tension is too low, the film edges can become loose, causing telescoping, where layers of film shift out of alignment with each other [4,14,86]. Additionally, if tension is too low, wrinkles may appear on the roll, resulting in a wavy, uneven surface.

**Figure 14:**
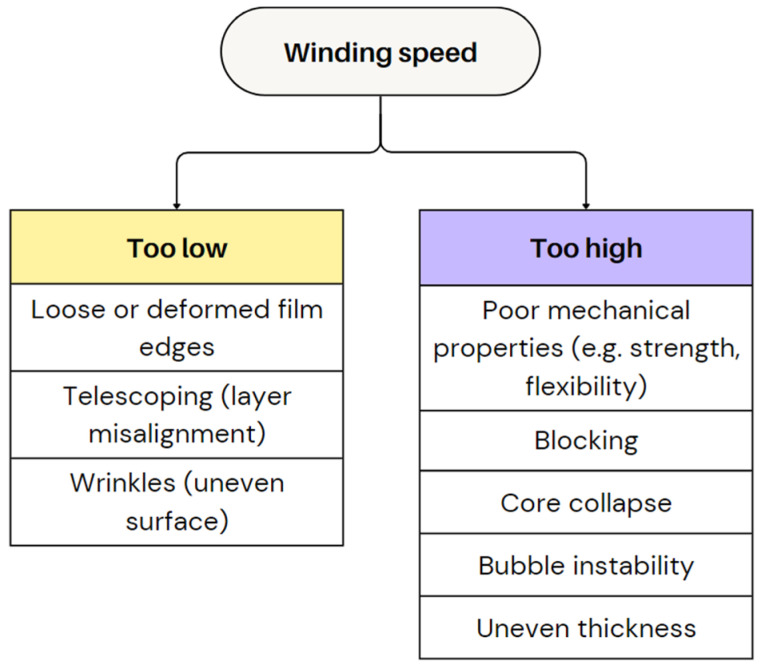
Overview of defects caused by improper winding speed (out-of-spec conditions).

A higher winding speed typically results in a faster rate of film accumulation, which can reduce stretch in the TD and potentially decrease the BUR, compromising film properties such as strength and flexibility [62]. Excessive winding tension can also lead to blocking or even core collapse, where the film becomes overly compressed around the core. If the winding speed is too high, the bubble may become unstable, leading to irregular thickness and a compromised final product [18]. Adjusting the pressure and alignment of the winder helps to avoid these technical issues, while introducing a chill roll can reduce wrinkling by stabilising the film temperature before it is wound [14]. The winding speed is generally set at a slightly higher speed than the nip roller speed so that the foil can roll up properly while there is some tension on it. Figure 14 illustrates the effects of improper winding speed and tension on film quality and roll formation.

#### 2.2.7. Equipment Settings and Design

The settings and design of the equipment also play a role in the quality of the film [14]. Wear and incorrect settings of components such as the extruder type, mounted die, cooling ring, and winding system can contribute to defects [14,64,80,87]. For example, a worn extruder or a damaged die opening can lead to uneven film formation [12]. An inefficiently functioning cooling ring may cause an unstable bubble and variable thickness [14,80]. The design of the die opening significantly impacts film thickness and width; an uneven die opening can cause thickness variations, while more modern adjustable dies allow for precise control of thickness and help maintain melt uniformity [14]. The flow channel design also plays a role in ensuring even melt distribution, promoting film consistency. The configuration and efficiency of the air ring are equally crucial because they determine cooling rate and bubble stability [76]. Uneven cooling may result in deformations and thickness inconsistencies, but advanced air ring designs with dual openings or tandem configurations can improve stability and uniformity.

Additionally, environmental factors like drafts in the factory can disrupt bubble cooling, causing uneven thickness, while temperature and humidity can impact film properties such as shrinkage and blocking [14]. Managing these factors by enclosing production spaces or controlling ambient conditions can further reduce defects.

#### 2.2.8. Interdependence of Process Variables

The interdependence of process variables in blown film extrusion requires some knowledge and has led to improvements in control techniques. Variables such as extrusion temperature, cooling rate, winding tension, nip roller speed, and screw speed all affect the bubble geometry and, hence, film properties. Adjusting one variable, like increasing nip speed to reduce film thickness, also affects FLH and the lay flat width. Operators therefore ideally need to understand how each adjustment affects multiple properties to effectively control film quality [33].

In conclusion, proper tuning and maintenance of the process parameters are essential to produce defect-free, high-quality films. Each processing condition must be optimised based on the specific polymer type and desired film properties, balancing these variables to ensure the film product meets the required standards for appearance, performance, and durability. As explained in the next subsection, specific points of attention can be put forward.

### 2.3. Material-Related Causes of Defects

In the plastic film blowing process, the quality of the base material plays a major role. The properties of the plastic, such as the chemical structure of the polymer and the amount and type of additives or NIAS, determine the processability of the material and the structural properties of the final film. If the choice of material is not optimal, it can lead to defects such as reduced transparency, uneven thickness, wrinkles, or poor sealability.

Influence of molecular structure and material properties. The material choice plays an important role in determining not only the process efficiency but also the quality of the film. Defects are strongly influenced by the average MW, MWD, and branching degree [5]. Below, important molecular properties of the material are discussed, and how their selection affects the film blowing process.

(i) Molecular weight or molar mass

The MW of polymers plays a critical role in determining both the processing behaviour and final properties of the films. High-molecular-weight polymers generally provide better film strength, due to enhanced chain entanglement [88,89]. However, crosslinked or high-molecular-weight polymers can cause fish eyes. These rubbery gels pass through screen packs unfiltered, disrupting the uniformity and optical quality [4]. Moreover, polymers with high MW are more prone to melt fracture, a defect that occurs at high shear rates, causing surface irregularities and a rough film appearance [4,89]. This tendency increases due to greater chain entanglement, which enhances resistance to flow and makes the polymer more susceptible to melt fracture [5].

The average MW of a polymer also has a crucial impact on its viscosity, which influences the processing behaviour and, in turn, shapes the film properties [5,89]. For film blowing applications, high viscosities are generally preferred, as they maximise the polymer’s deformation capacity within the specification range [4,5]. Higher average MW results in increased viscosity, making the polymer more resistant to flow under heat and pressure. Consequently, this requires greater processing effort, including higher drive power and adapted screw designs to ensure stable extrusion [4]. Furthermore, high MW materials promote sufficient melt strain hardening, which stabilises the melt curtain once the film is drawn, reducing the risk of defects [5,90]. While this resistance enhances mechanical strength and stability, it also makes it more difficult to process. Conversely, lower average MW corresponds to a lower viscosity, allowing the polymer to flow more easily but potentially reducing mechanical integrity, lowering the film’s strength, tear resistance, and durability [4,89]. Despite that a lower viscosity simplifies processing by reducing resistance to flow, an overly low viscosity can lead to a narrower processing range and insufficient melt strength, making the film more prone to instabilities during processing [4,91].

**Figure 15:**
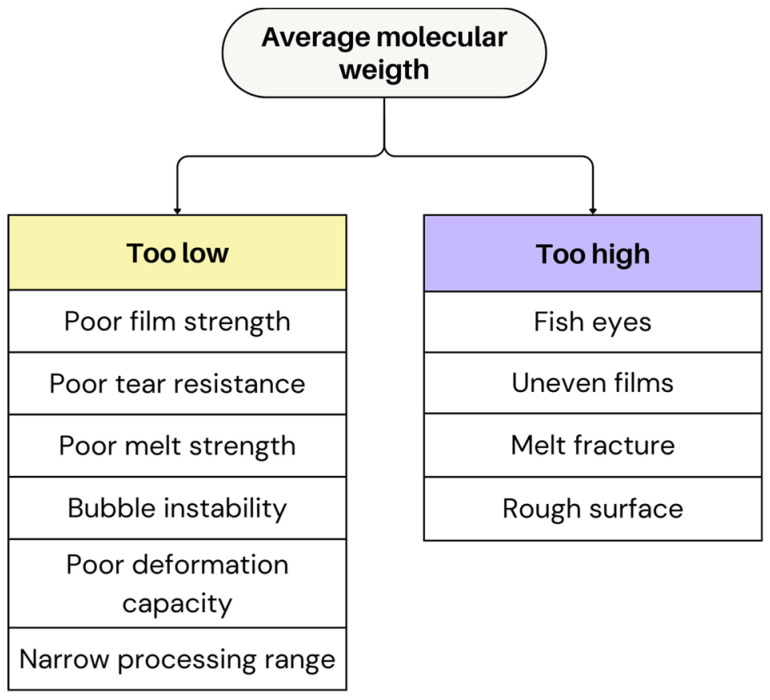
Overview of defects caused by improper average molecular weight (out-of-spec conditions).

In addition to its influence on the production, the average MW has a decisive impact on the final film properties. High MW grades of HDPE, for instance, are essential to achieve films with desirable stiffness, impact resistance, and a matte surface, while lower MW grades produce glossy but weaker films with a higher tendency to split under stress [4]. In applications such as machined direction oriented (MDO) films, the presence of lower MW fractions can enhance the (post-orientation) Young’s modulus, although this comes at the expense of reduced strength [88]. Conversely, higher MW improves molecular entanglement and, consequently, mechanical strength and impact resistance [4]. For film blowing applications, selecting the optimal average MW is crucial for achieving a high-quality film with desirable mechanical properties and processability [5]. Figure 15 demonstrates the consequences of both low and high average MW polymer on film characteristics and the occurrence of defects.

(ii) Molecular weight distribution

Polymer molecular weight (Mw) and distribution critically influence melt strength, viscosity, and bubble stability in film blowing. Higher M_w_ increases melt strength and extensional viscosity, improving bubble stability and allowing higher blow-up ratios, but reduces processability due to high viscosity [68,69]. Low-Mw polymers flow easily but produce weak bubbles prone to necking.

**Figure 16:**
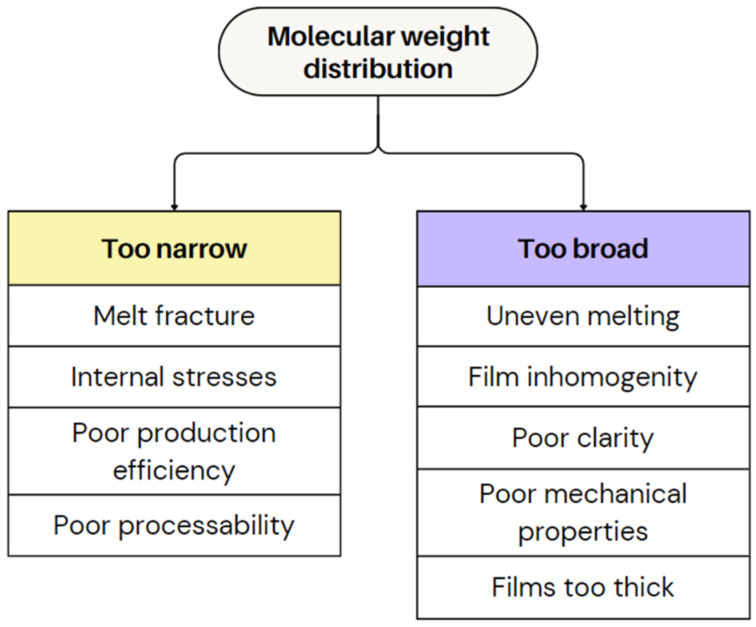
Overview of defects caused by improper molecular weight distribution (out-of-spec conditions).

The width of the MWD, i.e., dispersity, can also influence defects in plastic films, like the intensity of melt fracture. A broader distribution allows for better stress distribution, reducing the likelihood of these irregularities [5,44]. A wider MWD causes stronger shear thinning, leading to lower viscosity at high shear rates, potentially increasing the production efficiency. However, it can also cause uneven melting temperatures, affecting film homogeneity and surface quality [5]. Moreover, a broader MWD can improve impact strength, due to enhanced chain entanglements from higher MW fractions but may reduce tear resistance because of lower MW chains [89]. Optical clarity also tends to decrease as broader MWD increases haze and reduces gloss, likely due to more surface irregularities [89]. As a result, a high MWD can cause inconsistent processing behaviour, reduced film strength, lower optical clarity, and difficulty in producing thin films. Figure 16 presents the impact of varying MWD on the film quality.

LDPE blown film grades, typical weight-average molecular weights range 80,000–250,000 g/mol, with dispersity (Mw/Mn) of 4–7, balancing processability and melt strength [14].

(iii) Branching level

Not only the average MW and the MWD shape but also polymer chain architecture, such as the degree and length of branching, play a critical role in the film quality. Branching density in polymers strongly affects melt strength, bubble stability, and drawdown behaviour.

Long-chain branching (LCB) causes stronger shear thinning, lowering viscosity at high shear rates and therefore improving processing efficiency. It also improves the bubble stability but can increase the relaxation time, which may reduce the film strength [4,5,73]. In addition, LCB can cause uneven melting, impacting film homogeneity and surface quality [5,73].

**Figure 17:**
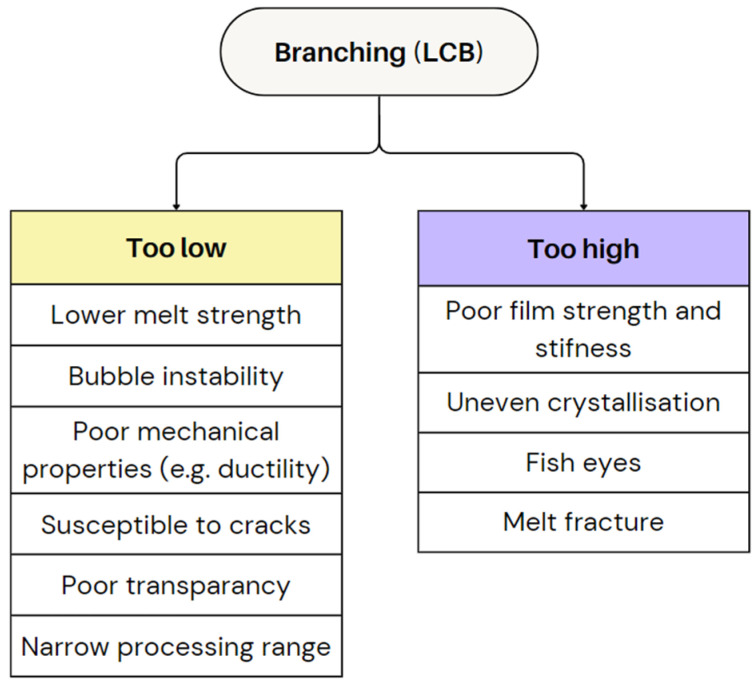
Overview of defects caused by improper branching (out-of-spec conditions).

Branching, particularly LCB, also influences crystallisation. Higher branching levels tend to have slow crystallisation rates, leading to a less ordered molecular packing that reduces transparency but improves flexibility and toughness [4,89]. This is due to the scattering of light by the denser, more organised crystal structures in the film, which diminishes clarity [4]. For instance, LDPE, with substantial LCB, shows lower crystallinity compared to LLDPE, enhancing ductility and tolerance to broader processing conditions [5]. This makes LDPE more resistant to cracks and fractures, improving toughness, though at the expense of stiffness and tensile strength [64,89]. While a higher LCB may reduce clarity by introducing surface irregularities, certain LDPE grades achieve good optical properties without compromising strength [64]. In contrast, LLDPE, with short-chain branching (SCB), offers greater stiffness and tensile strength but is more prone to defects like cracking and has a narrower processing window [5,73]. Also, the type of comonomer used in LLDPE significantly affects the final film properties [5]. Overall, the degree and nature of branching significantly affect the rheology, deformation behaviour, and crystallisation during film blowing, shaping the final film morphology and properties [5]. Figure 17 provides an overview of the challenges and performance issues associated with both low and high branching levels.

For LDPE, typical long-chain branching density is 0.02–0.05 branches per 1000 carbons, providing a balance between processability and mechanical performance [14].

#### 2.3.1. Polymer Degradation

Polymer degradation is a complex process that significantly impacts the quality and properties of plastics, including blown films [92]. Different types of degradation occur depending on the polymer type and environmental conditions, leading to defects and reduced performance. Polymer degradation occurs through various mechanisms, each with its unique causes. Yet, the outcomes are often similar, affecting the mechanical strength, optical clarity, and barrier performance of blown films [93]. It commonly results in discolouration (e.g., yellowing), increased brittleness, and reduced tensile strength [56].

The primary cause of material degradation is heat, which is already discussed in Section 2.2.1 [56,58]. Thermal degradation occurs in case high temperatures during processing or use cause chain scission and/or crosslinking. This leads to discolouration, loss of mechanical strength, and the formation of volatile decomposition products. This often results in brittleness and increased susceptibility to tearing [92,93,94,95]. Thermo-oxidative degradation, driven by heat and oxygen exposure, accelerates oxidation and chain scission, causing embrittlement, surface erosion, and yellowing [92,93,94]. These phenomena contribute to defects such as cracks and brittle areas, reducing film durability [82].

(Thermo-)mechanical degradation results from physical stress such as shear forces, impact, or friction, leading to chain scission, radical formation, and MW reduction [92,93,96]. This weakens mechanical properties, causing cracks and surface defects, especially under high-stress conditions [92,93,94].

Chemical degradation occurs if polymers are exposed to aggressive substances such as acids, bases, and solvents. This leads to structural changes, chain scission, and mechanical property loss [94,95]. Chemical degradation weakens the material, making it more prone to failure, especially in harsh environments [94,95]. Additionally, the use of certain nanofillers in polymer composites can accelerate degradation. Some nanofillers promote radical formation, increasing the degradation rate and negatively affecting the mechanical stability of the films [94].

Photodegradation, caused by UV exposure, breaks polymer chains (more during use), which weakens mechanical properties, causes colour changes, and leads to surface erosion. This reduces barrier performance and shortens the lifespan [93,95]. Hydrolytic degradation occurs if polymer chains react with water, particularly in materials containing hydrolysable bonds like esters and amides. This process typically reduces the average MW and mechanical integrity, making the films weaker [93,94]. For completeness it is mentioned here that bio- or enzymatic degradation is initiated by microbial activity for which enzymes break down polymer chains into smaller molecules. While this process is beneficial for biodegradable plastics, it is undesirable for conventional polymers because it can lead to material deterioration and defects [93,96].

These degradation types rarely occur in isolation but often interact and accelerate each other. For instance, thermal oxidation increases sensitivity to UV degradation, while mechanical stress can enhance hydrolytic degradation [93,95]. Additionally, polymer impurities and additives influence degradation rates [94]. One of the most concerning outcomes of polymer degradation is the formation of microplastics. Physical and chemical degradation processes break down polymers into microscopic particles, which accumulate in the environment, posing risks to ecosystems and human health [93,95].

Recycling also contributes to material degradation because plastics, for instance, undergo thermal and mechanical degradation during processing. The repeated melting and extrusion of polymers can gradually break down polymer chains, weakening the material over time [56,58,93]. Careful monitoring is needed to ensure that degradation does not significantly impact the film properties [57,58]. Additionally, recycled materials often contain more NIAS, which can further accelerate degradation. These NIAS, such as foreign particles or incompatible polymers, disrupt the polymer structure, creating stress points that weaken the material. If the polymer mixture includes polymers with lower processing temperatures, they are more prone to degradation, which increases brittleness and reduces overall strength [93].

**Figure 18:**
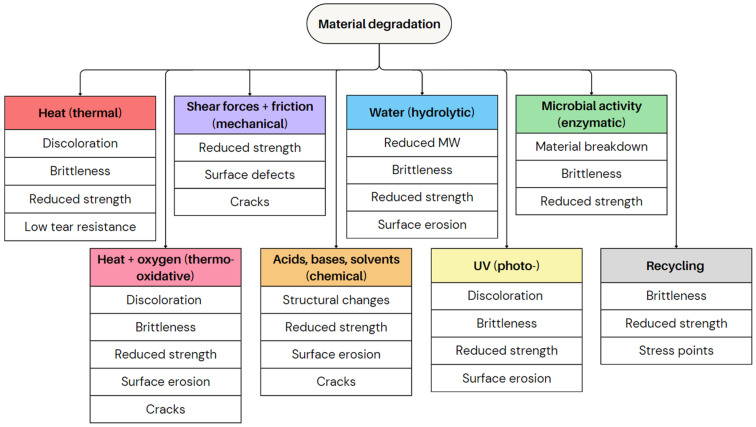
Overview of defects caused by degradation (out-of-spec conditions).

Figure 18 depicts the defects and quality limitations resulting from different types of polymer degradation. Understanding these degradation mechanisms is essential for improving polymer stability, and extending the lifespan of the film applications [95,96]. The development of stabilisation methods, such as UV absorbers, radical scavengers, and antioxidants, helps to mitigate degradation and maintain the quality of polymer films over time [94]. However, the ongoing challenge is finding a balance between performance, durability, and environmental impact to optimise polymer applications while minimising long-term degradation effects [93].

#### 2.3.2. Material Purity and Composition

**Figure 19:**
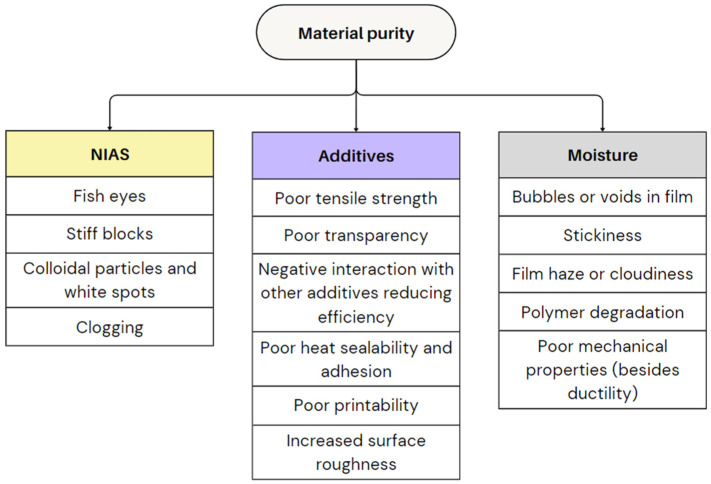
Overview of defects caused by improper material purity (out-of-spec conditions).

Polymer purity is another key factor that significantly impacts the film blowing process. Contaminants like inorganic NIAS or the inclusion of different polymers, typically present in recycled plastic, can lead to unpredictable behaviour, creating heterogeneous blends that can disrupt the film blowing process. Additives are often applied in substantial amounts to improve material properties or limit the deterioration of properties upon using recycled materials. However, their interaction with the base polymer can still compromise the stability of processing conditions. Additionally, moisture content in the raw material can introduce further complications, especially for hygroscopic materials, impacting film quality and consistency. The following subparts explore these factors in greater detail. Figure 19 illustrates the defects caused by improper material purity and provides a visual representation of the potential quality issues.

(i) Non-intentionally added substances

NIAS present in raw materials or introduced during the extrusion process can lead to several defects that negatively impact the quality of the film. One of the most common defects caused by contaminants is the formation of fish eyes. In addition to fish eyes, NIAS can also lead to other defects, such as stiff blocks in the film and colloidal particles or white spots. Stiff blocks can form if materials are not properly melted or when filter elements break, while colloidal particles and white spots are often the result of impurities in the raw materials or breakdowns in the filter [4,14]. Overheating of the material during extrusion can also lead to decomposition of the products that contribute to these defects. Moreover, NIAS can cause clogs in the die or other parts of the extrusion line, leading to reduced output and further defects in the film quality [4,14].

To prevent defects caused by NIAS, it is crucial to maintain strict control over the quality of raw materials. Testing films made from raw materials can help to detect potential gel formation before production begins [4]. The addition of additives such as lubricants and anti-blocking agents may help to reduce gel formation, although it cannot fully eliminate the issue [14,97]. Regular maintenance and cleaning of the extruder and die inlet points are essential to remove contaminants and ensure smooth production [4,14]. Additionally, optimising extrusion conditions, such as starting with a stable melt and adjusting the melt temperature and shear rate profile, can help to minimise the formation of fish eyes [4,14]. The use of filters or screen packs also plays a very important role in removing contaminants from the melt, although care must be taken to ensure filters do not break and cause further defects [4,14].

(ii) Additives

Additives play a crucial role in the quality of polymer films and the occurrence of defects. They are commonly used to enhance processability, increase output, and prevent issues such as melt fracture [45,98,99,100]. By reducing shear stress and promoting smoother polymer flow, additives help to optimise film production. Polymer processing aids (PPAs), such as hyperbranched polymers (HBPs) and fluoroelastomers, are particularly important [45,98]. These substances reduce frictional resistance, preventing defects like melt fracture while improving processability [45,98]. PPAs migrate to the surface, forming a lubricating layer between the polymer and metal surfaces, which decreases friction [45,98,100]. HBPs also tend to migrate to the surface, forming a lubrication layer that can significantly increase processing speed by at least 40% [98]. By lowering shear stress at the die wall, a higher output can be achieved and die buildup can be prevented [45]. In addition to PPAs, other additives are essential for the film blowing process. Anti-blocking agents, such as silica, prevent film layers from sticking together during winding [45,99]. Slip agents, like fatty acid amides, reduce the coefficient of friction, ensuring smoother movement of the film through processing equipment and mitigating blocking, in particular during winding and unwinding [99]. Furthermore, additives such as UV stabilisers and antioxidants improve the stability of the polymer during processing and use, preventing defects due to degradation [45,100].

While additives offer many advantages, they can also negatively impact film properties. The use of PPAs, for example, can reduce gloss and transparency, likely due to their effect on the crystallinity [101]. PPAs may also alter mechanical properties, sometimes leading to reduced tensile strength [98,99,102]. Furthermore, they can interact negatively with other additives, such as anti-blocking agents or fillers, reducing their effectiveness and extending the time required to eliminate melt fracture [45,100]. Another concern is additive migration, particularly with PPAs, which can affect adhesion, printability, and heat sealability. However, at low concentrations, migration is often negligible [45,98]. Anti-blocking agents increase the surface roughness, which helps prevent film adhesion but may also reduce clarity [99].

To produce high-quality films with minimal defects, careful selection and optimisation of additives are crucial. A balance must be found between the benefits of improved processability and potential negative effects on mechanical, optical, or functional properties. Specific solutions to mitigate defects include the use of masterbatches, which allow additives to be incorporated separately, minimising unwanted interactions [45]. The addition of interfacial agents, such as polyethylene glycol (PEG) and polycaprolactone (PCL), can help to reduce negative interactions between PPAs and anti-blocking agents [45]. Mechanical treatment of the polymer melt can also be applied to decrease melt elasticity and reduce film haze [45,51].

(iii) Moisture

Water contamination in the polymer can lead to defects in the final film. While it may not always be the primary cause of defects, it often contributes in combination with other factors [4,14]. During the film blowing process, any moisture trapped in the polymer can vaporise, causing bubbles or voids within the film. Foaming and bubble formation occur in case excessive moisture in the melt leads to steam formation at high processing temperatures. This results in optical imperfections, such as haze or cloudiness, which negatively affect the film’s clarity. To prevent this, precise control and optimisation of the material’s moisture content, as well as adjustments to the temperature profile to avoid sudden vaporisation, are essential [61]. Additionally, the presence of water can promote polymer degradation, further weakening the film’s mechanical properties and reducing its overall quality [103]. Surface defects, such as stickiness, can arise due to high moisture content in the melt. These technical issues can be addressed by pre-drying the material to lower moisture levels [61].

Humidity also significantly affects the properties. A high humidity acts as a plasticiser, increasing the film’s extensibility while decreasing its tensile strength, as water makes the polymer chains more mobile [104]. In contrast, a low humidity can make films more brittle, reducing elongation at break [104,105]. This effect is particularly noticeable in hydrophilic biopolymers, which readily absorb water [99]. The mechanical properties of these films are also strongly influenced by ambient humidity [104,106]. Furthermore, the humidity can impact biodegradability and the degradation rate, because moisture accelerates hydrolysis in susceptible polymers, especially under alkaline conditions [105]. Plasticisers like glycerol can mitigate the adverse effects of humidity, and enhance the film properties [106]. Additionally, the addition of urea helps to facilitate starch gelatinisation at low water contents, promoting the extrusion of uniform films [107]. Effective humidity control is crucial for consistent film quality during production and storage.

## 3. Sustainable and Advanced Film Types

In addition to conventional plastic films, recent years have seen significant research focused on developing new strategies and new materials to enhance film properties and improve durability. These innovations include the use of recycled and biodegradable materials, polymer blends, multilayer film structures, and mechanical treatments such as MDO stretching [26,108,109]. Although these technologies offer promising benefits, they also introduce specific material characteristics that require adapted processing conditions and are therefore associated with a higher likelihood of distinct defects [5,110,111]. To provide a clear and systematic discussion, each subsection below includes: (i) a brief introduction to the film type with its key material characteristics and product issues; (ii) the underlying causes; and (iii) feasible mitigation strategies.

### 3.1. Recycled Films

#### 3.1.1. Material Characteristics and Product Issues

Flexible packaging offers many advantages and is resource-efficient, but recycling rates remain low despite strict EU recycling targets for 2030 [112]. Post-consumer waste (PCW) typically contains a mix of unknown polymers (such as PE, PP, and polyethylene terephthalate (PET)) and non-polymeric materials (such as paper and aluminium), making separation and reprocessing difficult [22,113]. This leads to compatibility issues in case different plastics are mixed, weakening mechanical properties [22,114]. Incompatible polymers, like PET and PE, can cause phase separation, reducing the toughness and ductility of recycled materials [115]. PCW also contains NIAS such as inks, adhesives, coatings, and labels, which are difficult to remove and negatively affect colour and odour [22,113,114,115]. In addition, the presence of polar contaminants can make films hygroscopic, increasing moisture content to above 0.1–0.3 wt% and complicating processing [22,114]. Recycled material may also exhibit altered viscosity due to thermal, mechanical, or UV-induced degradation, which affects crystallinity and MFR and complicates film blowing [22,114].

Films made from recycled material are generally more susceptible to defects, because of the varying composition and contamination level of PCW. Typical defects include unmelts, specks, stripes and fish eyes, which create surface roughness and reduce mechanical performance [116]. Die drool, which occurs when molten material accumulates on the extrusion die, is also more frequent during processing of recycled polymers. Inconsistencies, NIAS, and insufficient mixing promote this buildup at the die lips, leading to poor surface quality, die lines, and reduced productivity [117,118,119]. These irregularities can disturb bubble stability, contribute to gauge variation, and reduce optical clarity. Recycled streams may also cause higher moisture levels, which can introduce further instabilities during extrusion [22,114].

#### 3.1.2. Causes

Most of the issues encountered in recycled films originate from the intrinsic heterogeneity of post-consumer waste. Mixed or incompatible polymers cause phase separation and weak interfaces. Contaminants and NIAS introduce colour defects, odour problems, and higher moisture uptake [22,114]. Degradation during previous cycles alters the melt rheology, leading to unstable film formation, reduced mechanical properties and poor optical quality. Filler agglomeration or poor dispersion can further disturb film uniformity, while sorting limitations make it difficult to obtain a predictable feedstock, especially in the case of multilayer packaging or black plastics [22,113,114,115]. The trend towards thinner films and the increasing use of more complex waste streams also intensifies problems such as die drool [117,118,119]. Die drool can reduce surface quality and productivity, particularly with thinner or multimodal films [119].

#### 3.1.3. Mitigation Strategies

Producing blown films from recycled materials requires adapted processing conditions, because the viscosity and melt behaviour of the material can change significantly due to thermal, mechanical, and UV-induced degradation during previous use cycles [22,113,114,115]. High heat and shear during re-extrusion alter the average molecular mass through branching, cross-linking, or chain scission, which modifies crystallinity, shrinkage and MFR [22,113,114,115]. To limit further degradation, it is often necessary to lower temperatures or reduce RT according to polymer type and equipment. Stabilising additives such as antioxidants, heat stabilisers, and UV stabilisers can also help maintain molecular structure [22,113,114,115].

**Figure 20:**
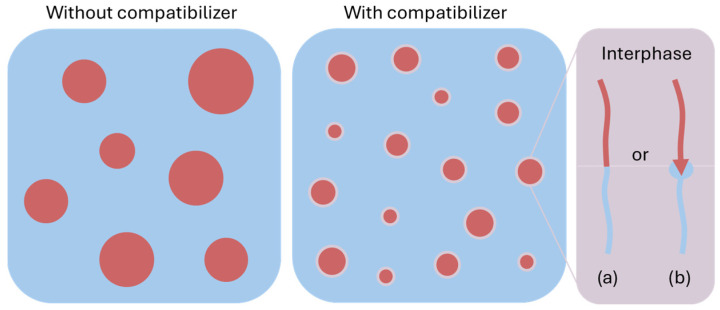
Schematic representation of the effect of compatibilization on polymer blends using (**a**) block copolymers and (**b**) reactive polymers. The arrow signifies the coupling of the reactive functional groups.

Compatibilizers improve the compatibility of polymer blends by interacting chemically or physically with both components, using reactive compatibilization or block/graft copolymers to stabilize the interface (Figure 20) [112,120,121]. They reduce phase separation, improve polymer embedding, and result in a more uniform film structure with better bubble stability, thinner films, and fewer thickness variations [112,121,122]. Compatibilization can also refine morphology; enhance transparency; and increase tensile strength, elongation at break (especially in the TD), strain hardening, and impact resistance [112,121,122]. However, excessive concentrations can lead to crosslinking and limit these benefits, so careful selection and use (typically less than 10–15% of the mixture) is crucial for effective mitigation [112,121]. Choosing a compatibilizer that bonds well with all polymers in the blend is also essential to ensure optimal performance.

Fillers can further modify film properties, with effects depending on type, size, concentration, and interaction with the polymer matrix [112,113,114]. They can enhance stiffness, surface hardness, melt strength, and film stability, and act as drying agents or improve interfacial adhesion and component dispersion [22,112,114]. Mineral fillers can reduce additive migration, and nanoscale fillers improve compatibility, particularly when combined with compatibilizers [22,112]. However, poor dispersion or agglomeration can harm mechanical and optical properties, reduce transparency (though minimally at <5% concentration), and increase density, which is generally undesirable [22,113].

Processing adjustments, such as die-geometry optimization or lubrication additives, help improve extrusion stability and reduce defects like die drool [119]. Melt filtration is another important step, since it removes inorganic NIAS and high-melting-point polymers that would otherwise lead to unmelts [22,113,114,115]. Improved washing, drying, and de-inking increase material purity and stability during extrusion. Because sorting remains a major bottleneck, better identification technologies such as digital watermarks, NIR, and AI-assisted recognition can provide a more consistent feedstock for film blowing [22,113,114,115]. Closed-loop recycling, for instance, where materials are reused in the same applications, ensures consistent product quality and a more sustainable lifecycle [22,115]. Lastly, design-to-recycling strategies, like using monomaterial multilayers, compatible polymers, and enabling easy separation, will enhance recyclability and support a circular economy.

Using materials like recycled LDPE, which crosslink rather than chain scission during degradation, preserves polymer integrity, yielding higher toughness, elongation, tensile strength, and good transparency [109,121,123]. Blends of recycled LDPE and LLDPE further improve mechanical properties due to synergistic crystallinity effects, though shrinkage may increase [123]. Similarly, recycled PP can be upcycled via reactive extrusion with peroxides to create long-chain branching, improving melt strength, strain hardening, and impact resistance, followed by extrusion with stabilizers to prevent degradation [124,125].

Over-engineering, in which extra material is added to compensate for reduced mechanical properties, is another option, although it may lead to higher plastic consumption [115]. Finally, and perhaps the most critical task, is increasing the demand for recycled materials and raising consumer awareness, encouraging proactive solutions rather than avoiding them [22]. A summary of common issues and defects in recycled films, including their causes and mitigation strategies, can be found in Table 2.

### 3.2. Biodegradable Films

#### 3.2.1. Material Characteristics and Product Issues

The production of films from natural, biodegradable polymers presents several challenges and processing and material difficulties due to their inherent material properties and sensitivity to processing conditions. Bio-based packaging must compete with commodity polymers in protection, preservation, and convenience while remaining ecological and cost-effective. Currently, bio-based films fall short, but ongoing research aims to enhance their mechanical, thermal, and barrier properties to replace conventional packaging [126]. One major issue is thermal degradation, because natural polymers are often more heat-sensitive than conventional plastics [127,128,129]. High temperatures and shear forces during extrusion can break down polymer chains, leading to reduced mechanical properties such as lower tensile strength.

Another common problem with biopolymers is the difficulty in film formation and the limited BUR [130]. The low melt strength of biopolymers like starch and polylactic acid (PLA) often causes instabilities, leading to films with uneven thickness and poor mechanical performance [127,130]. Brittleness and poor stretchability, particularly in PLA films, complicate processing and increase the risk of tearing [128,129,130]. The addition of modified biofillers such as chitosan and gum arabic, combined with crosslinking agents, can enhance flexibility and performance by strengthening intermolecular hydrogen bonding [130], although adding fillers may affect thickness and homogeneity, especially when modified fillers are used [129]. Agglomeration of fillers can also negatively impact film integrity [130].

The hygroscopic behaviour of certain biodegradable polymers, particularly starch, is another significant drawback. These materials readily absorb moisture, which alters their mechanical properties depending on ambient humidity [61]. Water molecules interact with the polymer chains via hydrogen bonds, leading to swelling, softening, and a reduction in strength and durability [131]. This sensitivity also causes poor barrier properties against gases, moisture and aromas, limiting applications in the food packaging sector. Many biodegradable polymers are inherently hydrophilic, allowing water vapour to pass through easily, compromising their effectiveness as protective films [132,133,134]. As a result, these materials struggle to provide adequate protection against water, oxygen and other small molecules.

Starch-based films are additionally affected by retrogradation, which causes starch molecules to recrystallise, resulting in brittleness and poor flexibility over time [132,135]. Variations in raw material quality pose further difficulties because the composition of natural polymers can vary depending on climate, geographic origin, and storage conditions [128]. Natural polymers are also prone to microbial and dust contamination during collection, transport, and storage, affecting film quality [127,128].

#### 3.2.2. Causes

Many of the issues observed in biodegradable films stem from the inherent molecular characteristics of natural polymers. Their sensitivity to heat and shear promotes thermal degradation during extrusion, resulting in chain scission and reduced molecular weight [127,128]. Low melt strength in polymers like PLA and starch leads to poor film-forming behaviour and instability during blowing [127,130].

Hydrophilic functional groups (e.g., hydroxyl groups) promote moisture absorption, causing swelling, softening and reduced mechanical stability [132]. These same groups facilitate diffusion pathways for water vapour and small molecules, which explains the generally poor barrier properties of biodegradable materials [132,133].

Filler-related problems originate from insufficient dispersion or agglomeration, which disrupts structural uniformity and can impair both mechanical and optical properties [127,130]. Variability in raw-material composition and contamination during handling further contributes to inconsistencies in processing and film quality [127,128]. Retrogradation in starch arises from molecular reorganisation and recrystallisation during and after processing. Processing temperature, residence time, plasticiser type, enzyme activity and storage conditions all influence the rate and extent of retrogradation [131,132,133,135].

#### 3.2.3. Mitigation Strategies

Several strategies exist to improve the processability and performance of biodegradable films. Thermal degradation can be limited by optimising screw configuration, temperature profiles and RT. Modelling tools (e.g., kinetic Monte Carlo simulations [136,137,138]) can support this optimisation. Plasticisers are frequently added to improve processability and reduce degradation. To address low melt strength, LCB agents or blending with other polymers can help stabilise the melt and improve film quality [127]. Brittleness and poor stretchability, particularly in PLA, may be mitigated by incorporating plasticisers or blending with more elastic biopolymers such as polyhydroxybutyrate (PHB) [127,129]. Fillers such as chitosan or gum arabic, combined with crosslinking agents, can improve flexibility, although proper dispersion is crucial. Issues with agglomeration can be resolved by using non-ionic surfactants or modifying filler surfaces through coating, blending or grafting to strengthen hydrogen bonding across the matrix [128,130].

Retrogradation can be reduced through chemical, enzymatic or physical modification of starch. Chemical modification alters molecular structure through plasticisation, blending or specific chemical treatments [132]. Blending starch with other biopolymers such as polyvinyl alcohol (PVA) can enhance both mechanical and barrier properties [133]. Enzymatic modification using β-amylase influences gelatinisation and retrogradation behaviour. Physical modification techniques, including heating, extrusion, reactive extrusion, electrospinning and nanotechnology, also contribute to improved stability [134].

To mitigate moisture sensitivity, increasing crystallinity can reduce water absorption, although with some loss of flexibility. Chemical modifications such as esterification and etherification can enhance water resistance, and hydrocolloid additives can further enhance water resistance [135]. Barrier properties can also be improved through hydrophobic coatings or by applying metal or metal-oxide layers, although the impact of such metallisation on biodegradability must be carefully considered. Another approach is molecular-structure design, in which suitable monomers are selected to modify the polymer architecture and reduce permeability [133]. Compatibilizers for biodegradable blends can help balance oxygen and water-vapour barrier properties with mechanical strength, improving phase integration without compromising performance [132].

Advanced strategies include reducing barrier layers to the nanoscale. Nanocomposites based on polyvinyl alcohol (PVOH), polyglycolic acid (PGA) or polyhydroxyalkanoates (PHA) can provide highly effective oxygen-barrier performance [133]. Incorporating micro- or nanofibrils can also create a denser internal structure, increasing the diffusion path length for gases and moisture and significantly improving barrier behaviour [132]. Multilayer coextrusion using well-selected biodegradable components is another effective solution, enabling the combination of complementary properties across layers [133]. Despite these limitations, advancements in formulation and processing are steadily improving the viability of biodegradable films for packaging applications. Continued innovation in material selection, additive design and processing technologies will be essential to enhance performance and scalability. Table 3 gives an overview of the typical difficulties, root causes, and solutions related to biodegradable polymer films.

### 3.3. Blended Polymer Films

#### 3.3.1. Material Characteristics and Product Issues

Film blowing using polymer blends presents various processing challenges, primarily due to differences in the physical and chemical properties of the blended materials [139,140,141]. Nevertheless, polymer blending also offers significant advantages, such as enhanced mechanical properties, improved barrier performance, and cost efficiency [140,141]. As blends increasingly incorporate recycled or bio-based materials, understanding the origin of typical defects becomes even more important to ensure stable processing and consistent film quality.

One common issue is poor interfacial adhesion or polymer incompatibility, stemming from the thermodynamic immiscibility of the components and substantial differences in molecular properties [108,140,141,142]. Due to the long molecular chains, the entropy of mixing is inherently low, making phase mixing thermodynamically unfavourable unless strong favourable interactions are present [142]. As a result, the phases remain poorly bonded, leading to diminished mechanical strength and barrier performance in the final film [108,140]. Agglomerates and heterogeneities may form due to insufficient mixing of immiscible polymers, component incompatibility, or uneven distribution of compatibilizers. This can also reduce film transparency because light scatters at phase interfaces, especially when refractive indices differ significantly [143].

Inconsistent mechanical properties can also occur, as some areas of the film may be more brittle while others are more flexible due to phase separation [140,141]. Uneven thickness and processing instabilities can occur when differences in polymer rheology result in non-uniform stretching during film blowing. Low melt strength and low elongational viscosity of certain polymers can further contribute to bubble instability [61,139]. Another difficulty is thermal degradation and poor heat stability, particularly when blended polymers have different degradation temperatures or a narrow processing window [139].

#### 3.3.2. Causes

The main cause of most issues in blended films is the thermodynamic immiscibility of polymers: differences in polarity, MW, crystallinity, and surface tension make interfaces weak and incompatible [140,141]. This immiscibility promotes phase separation, which reduces adhesion and creates mechanical and optical defects. Heterogeneities and agglomerates originate from insufficient mixing, poor dispersion, or uneven compatibilizer distribution, especially in blends containing immiscible polymers [140].

Mechanical inconsistencies arise from non-uniform phase morphology, resulting in local brittleness or excessive flexibility [140,141]. Differences in rheology, including melt viscosity and melt strength, lead to non-uniform deformation of the film during blowing, contributing to thickness variation and bubble instability [139,144]. Even when polymers are individually stable, strong viscosity mismatch can still cause instabilities. Thermal degradation can occur because components degrade at different temperatures. Moisture, oxygen, and the presence of degradable components further aggravate thermal instability [139]. Moreover, light scattering at phase interfaces of the different blend materials explains the loss of film transparency when refractive indices differ [143].

#### 3.3.3. Mitigation Strategies

Compatibility issues can be reduced by adding compatibilizers, such as adding reactive compatibilizers that promote chemical bonding or entanglement at the interface. Additionally, block copolymers can facilitate specific interactions, such as hydrogen bonding and dipole–dipole interactions, further enhancing adhesion between blend components [140]. Proper selection and optimisation of compatibilizer concentration is essential to avoid excessive reactions or gel formation [140]. Improving mixing and dispersion through twin-screw extrusion, melt-blending procedures, and optimised compatibilizer distribution helps reduce heterogeneities and agglomerates [140,141]. Selecting miscible polymers or using compatibilizers can also improve transparency by promoting finer phase dispersion [140,142].

Mechanical inconsistency can be reduced by optimising blend ratios, ensuring proper compatibilization, and refining blending and processing conditions [141]. To address rheological mismatches and bubble instability, polymers with higher melt strength can be incorporated. Compatibilizers that increase melt viscosity and elasticity, for example, through crosslinking or LCB, may also stabilise processing, although excessive concentrations may cause gel formation. Adjusting processing parameters (temperature profile, screw speed, BUR, TUR) further improves bubble stability. In some cases, multilayer architectures with a supporting polymer layer can enhance stability during film blowing [139]. For thermal degradation and heat sensitivity, careful control of temperature, moisture and blend composition is necessary. Selecting PPAs that improve thermal stability can also help. Ultimately, defining an optimal processing window is essential to minimise thermal or mechanical failure [139]. Table 4 summarizes the typical difficulties, root causes, and solutions related to blended polymer films.

### 3.4. Multilayer Films

#### 3.4.1. Material Characteristics and Product Issues

**Figure 21:**
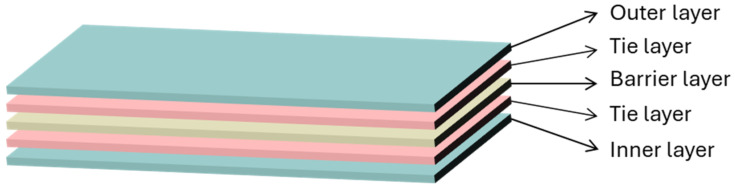
Example of a simple multilayer film structure.

Multilayer or coextruded films are widely used for their ability to combine various mechanical, barrier, and optical properties that cannot be achieved with monolayer films, including blended ones. By layering different polymers (or polymer blends), it becomes possible to create films with tailored functionalities for specific applications, such as food packaging with a high vapour barrier [22,134,145]. However, while multilayer structures offer significant advantages, they also introduce a range of technical difficulties and potential defects that complicate both production and end-use performance. Figure 21 presents a conceptual drawing of a multilayer packaging structure, highlighting the main functional layers.

One of the most common issues in multilayer films is curling [146,147]. Curling refers to the tendency of films to warp or roll up, often due to asymmetrical layer structures. This defect is primarily caused by differential shrinkage between the various plastic layers once the film cools during the extrusion process [146,147]. Differences in crystallinity, thermal expansion coefficients, and residual stresses developed during processing lead to imbalanced internal stresses that force the film to curl. Materials like nylon and ethylene vinyl alcohol (EVOH) are known for strong curling behaviour due to their high crystallinity and shrinkage [146]. Interfacial instability is another significant issue and occurs at the boundaries between different polymer layers during coextrusion [148]. These instabilities can lead to wavy or distorted interfaces that compromise the appearance and mechanical integrity of the film. The root cause is often a mismatch in melt viscosities and elasticities of adjacent layers, particularly at high throughput rates, which results in non-uniform flow and interface deformation [147,148].

Layer breakage, or layer scission, may also occur, particularly when very thin layers are used to minimise costs or maximise barrier performance. High shear stresses inside the die can cause these thin, low-viscosity layers to rupture, forming discontinuous domains instead of continuous films [148]. Poor adhesion between layers is another major processing issue, often leading to delamination, especially if the film is subjected to mechanical stress or thermal cycling [148]. This typically results from the lack of chemical compatibility between polymer chains of adjacent layers, such as polyolefins with EVOH or polyamide (PA) [22,147]. Achieving uniform layer thickness around the circumference of the bubble is also a technical difficulty. This is especially challenging when adapting flat-film technologies, such as feedblocks and layer multipliers, to the circular geometries required for blown films [22]. Melt fractures and weld lines that form when melts merge in the circular die can lead to non-uniformities in layer thickness, affecting both mechanical properties and appearance [22,147].

Predicting the properties of these films also remains a technical limitation [22,147]. The final characteristics, such as gas permeability and bending stiffness, cannot be easily estimated by summing the properties of individual monolayer films [22,145,148]. The actual performance is influenced by the film’s multilayer structure, including layer thickness distribution, interfacial adhesion, and the thermal and stress history experienced during coextrusion and cooling [146,147]. Finally, the complex multilayer structure poses serious challenges for recycling. Because multilayer films typically consist of different polymers that are chemically incompatible, separating them for recycling is technically difficult and often economically unfeasible. Therefore, many multilayer films are classified as non-recyclable and end up in landfills or incineration facilities [22,122].

#### 3.4.2. Causes

The primary causes of issues in multilayer films are closely linked to the material properties and structural characteristics of the layers. Curling arises from differences in crystallinity, thermal expansion coefficients, and residual stress among the polymer layers, which produce uneven shrinkage during cooling [146,147]. Interfacial instability occurs when adjacent layers have mismatched melt viscosities and elasticities, particularly under high throughput conditions, disrupting laminar flow and causing wavy or distorted interfaces [147,148]. Layer breakage is typically the result of high shear stress acting on thin, low-viscosity layers as they pass through the die, leading to scission or the formation of droplets instead of continuous films [148]. Poor adhesion between layers is caused by chemical incompatibility between certain polymers, such as polyolefins combined with EVOH or PA, which prevents strong interfacial bonding [22,147]. Non-uniform layer thickness is often caused by uneven melt distribution, weld lines, or melt fractures that occur when multiple polymer melts merge in a circular die [22,147]. Predicting film properties is challenging because performance depends on the multilayer architecture as a whole, influenced by interfacial forces, layer distribution, and the thermal and mechanical history during processing [146,147]. Finally, recycling difficulties stem from the combination of chemically incompatible layers, making separation technically complex and economically unfeasible [22,122].

#### 3.4.3. Mitigation Strategies

Various strategies can mitigate these multilayer-film issues. Curling can be reduced by balancing layer thicknesses, selecting polymers with compatible shrinkage behaviour, and adding modifiers such as ionomers to materials like EVOH to reduce the modulus without significantly compromising barrier performance [146]. Increasing cooling rates and lowering processing temperatures can also help minimise differential shrinkage [147]. Predictive modelling, based on beam theory and pressure-volume-temperature (PVT) data, can further support curling mitigation by enabling better layer design [146].

Interfacial instability can be addressed by selecting polymers with more closely matched rheological properties and by controlling melt and die temperatures to adjust viscosities and stabilise flow [147,148]. Well-designed feed blocks and dies are necessary to promote laminar flow and prevent interfacial disturbances [148]. Layer breakage can be prevented through careful control of shear rates by adjusting output rates or die geometry, and by selecting materials with higher melt strength for the thinnest layers [147,148]. Moreover, poor adhesion can be improved using adhesive resins or tie layers, such as maleic anhydride (MAH)-grafted polyolefins or ethylene copolymers. These materials contain functional groups that interact chemically at the interface and significantly improve interface adhesion. The effectiveness of these tie layers usually depends on precise control of processing conditions [122,147]. To ensure uniform layer thickness, the annular die and internal flow channels must be carefully designed to ensure even polymer distribution. Close monitoring and control of MFR and pressures during extrusion are also essential to maintain uniformity [22,147].

Because multilayer-film properties are strongly influenced by the architecture and history of the film, it is essential to test final films under realistic conditions that simulate their intended use, rather than relying on theoretical calculations based solely on monolayer data [146,147]. Finally, improving sustainability requires the development of recyclable multilayer structures or compatible blends. Design-for-recycling principles, including monomaterial multilayers, compatible interfaces, and reduced complexity, can facilitate mechanical recycling and reduce environmental impact [22,122]. In Table 5, the typical technical issues, causes, and mitigation strategies related to multilayer films are compiled.

### 3.5. Machine Direction-Oriented Films

#### 3.5.1. Material Characteristics and Product Issues

**Figure 22:**
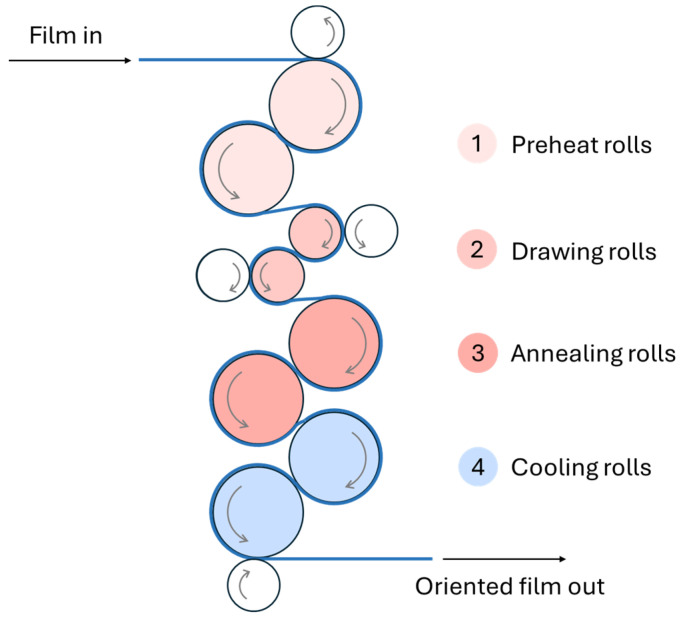
Schematic illustration of MDO process. The circles represent the MDO rolls with the corresponding function.

MDO films offer many advantages, such as improved tensile strength, stiffness, optical properties (in terms of haze and gloss), and water vapour transmission rate (WVTR) [24]. However, they also have several technical limitations and potential defects that can affect the quality and applicability of the final product [25,26,27]. Figure 22 outlines the MDO process, in which the polymer film is stretched longitudinally at an elevated temperature [149].

One of the primary processing issues is stretch resonance, which occurs due to uneven stretching or orientation [25,27]. This phenomenon is especially common with medium-molecular-weight high-density polyethylene (MMW-HDPE) resins that lack LCB, whereas high-molecular-weight HDPE (HMW-HDPE) generally performs well due to its strong molecular integrity [25]. Stretch resonance can lead to instability in film properties, causing fluctuations in film thickness and mechanical performance. Moreover, breakage or tearing during stretching is another processing issue, often resulting from the high stretching force required for proper orientation or the excessive density of the base material, leading to local stress concentrations and rupture [25]. Additionally, uneven draw or localised thinning (necking) can occur, leading to densely packed fibrils that hinder uniform deformation [25,27]. The enhancement of mechanical properties primarily in the MD can also result in lower TD strength if the process is not optimally controlled [25,27,149].

MDO films made from recycled materials are particularly sensitive to defects due to the stretching and orientation process [26,27]. Impurities and gel particles present in post-consumer waste (PCW) can become accentuated during MDO, leading to surface defects, point gels, fibre defects, and increased haze and a rougher texture [25]. A higher base material density can also increase fibrillation during material stretching [25].

#### 3.5.2. Causes

Stretch resonance arises from uneven stretching or asymmetrical orientation, particularly in MMW-HDPE resins that lack long-chain branching [25,27]. Breakage or tearing during stretching is caused by high stretching forces, excessive base material density, or local stress concentrations, while uneven draw or localised thinning occurs when the base material crystallinity is too high, forming densely packed fibrils that resist uniform deformation [25,27]. The imbalance between MD and TD strength results from the unidirectional nature of stretching, which enhances MD properties while reducing TD performance [25,27,149]. Defects in films made from recycled materials are caused by impurities, gels, or degradation products in PCW, which become accentuated during the stretching process, leading to surface roughness, haze, and fibrillation. High base material density further exacerbates fibrillation and uneven stretching [25].

#### 3.5.3. Mitigation Strategies

Mitigating defects in MDO films require a combination of material selection, process control, and careful handling of recycled content. Stretch resonance can be mitigated by using a proprietary processing aid with a low melting point [25]. Proper annealing, i.e., uniform heating before stretching followed by controlled cooling using thermal rollers, can prevent uneven film properties and warping by ensuring stress relaxation and dimensional stability [25]. Breakage and tearing can be minimised by optimising resin density and crystallinity, using additives to improve stretchability and reduce brittleness, and carefully controlling processing conditions to ensure even stretching [25]. Uneven draw and necking can be addressed by reducing crystallinity, preventing the formation of large crystalline spherulites, and fine-tuning the PE density to allow stable and uniform deformation [27,150]. The imbalance between MD and TD strength can be corrected by adjusting the MDO stretch ratio and carefully controlling processing temperature [25,27,149]. Using a relatively lower stretch ratio can improve the MD strength, while maintaining adequate TD strength, depending on the specific application [151]. To mitigate defects in recycled materials during stretching, crosslinking should be minimised, and the base material density should be modified by incorporating a higher fraction of, for instance, LLDPE (instead of LDPE) in the recycled material, since it primarily undergoes chain scission during degradation, or by reducing the fraction of recycled material [25,122]. Furthermore, the addition of a proprietary PPA can enhance clarity. A higher PCW quality, achieved through improved sorting, washing, or filtration, is critical to minimize these processing issues for MDO films made from recycled content. Table 6 summarises the common issues, their causes, and corresponding mitigation strategies for MDO films.

## 4. Conclusions

To summarise, film blowing technology is essential to produce a wide range of plastic films but faces significant challenges in defect prevention and quality assurance. This review shows that defects arise from various factors, including critical process parameters (such as temperature, nip roller speed, and cooling rate), fundamental material properties (such as average MW and purity), and the inherent complexities of both conventional and emerging technology film types. The complex process demands precise control over these interconnected factors to ensure consistent film quality. Common defects include bubble instability, gauge variation, wrinkles, melt fracture, surface irregularities like fish eyes, and optical issues like haziness or discolouration. Addressing these defects and processing issues is crucial for reducing material loss, ensuring required functional properties, and meeting growing sustainability and regulatory demands.

The future of the industry will be shaped by the transition to a more circular economy, requiring a shift towards recycled and biodegradable polymers. While these sustainable alternatives currently face limitations such as quality inconsistencies, degradation sensitivity, and limited barrier properties, innovations in material science, additives, and processing techniques will be key to overcoming these limitations [152]. Additionally, energy-efficient machinery, optimised material use, and waste reduction are essential for minimising the environmental footprint of the production [153].

This shift towards recyclable and biodegradable materials, such as PLA, PHA, and bio-based polyolefins, is accelerating as regulatory pressures and consumer demand push for greener packaging solutions [152,154,155]. Research into nanocomposites and polymer blending is also opening new possibilities for enhancing the mechanical and barrier properties of these natural materials, making them viable alternatives to conventional plastics [152,154]. However, the future of flexible packaging will depend heavily on improved recycling and sorting technologies to overcome issues like contamination and material degradation, which currently hinder large-scale adoption [22].

Technological innovations in extrusion technology, including model-based design, MDO stretching, automated control systems, and material optimisation, are expected to enhance film quality and process control while reducing environmental impact [12,153]. Polymer blends and multilayer films will continue to optimise performance and combine diverse functionalities, but success will depend on precise material compatibility and defect prevention [152,155]. The integration of “design-for-recycling” principles, especially in multilayer packaging, will be crucial in improving recyclability at the end of the product’s life cycle.

Finally, the integration of AI and big data analytics will enable intelligent production monitoring and defect detection, while automated production lines will improve process stability and reduce reliance on manual labour [14,153]. Additionally, enhanced process modelling and simulation techniques will help manufacturers better understand the relationship between material properties, processing conditions, and final film quality, enabling better control and scalability from laboratory to industrial production [80,156,157]. Overall, continued interdisciplinary collaboration and innovation across material science, engineering, and data technologies will be essential to drive sustainable and high-quality advancements in film blowing technology.

## Figures and Tables

**Table 2:** Technical issues, causes, and mitigation strategies in recycled films.

Technical Issues	Causes	Mitigation Strategies
Quality loss	NIAS (mixed polymers, inks, adhesives, …), degradation	Improved sorting and washing, melt filtration, compatibilizers, using virgin materials, blends (LDPE/LLDPE, PP upcycling), over-engineering [22,109,112,113,114,115,120,121,122,123,125]
Die drool	Material buildup on die (typically NIAS)	Modifying die geometry, lubrication additives, improved washing and sorting, using virgin materials [117,118,119]
Processing issues	Viscosity changes, degradation, melt temperature changes	Process optimization, additives (such as stabilizers and antioxidants), better sorting [113,115]
High costs	Recycling inefficiency, virgin plastic competition	Better recycling technologies, closed-loop systems, promoting recycling through incentives, design-to-recycling [22,113,115]

**Table 3:** Technical issues, causes and mitigation strategies in biodegradable films.

Technical Issues	Causes	Mitigation Strategies
Low quality	Thermal degradation (heat sensitivity)	Optimizing screw configuration, temperature, and RT; incorporating plasticisers [128,129,130]
Poor film formation and instability	Low melt strength, brittleness	Incorporating plasticisers; blend with (modified) biopolymers [128,130,135]
Retrogradation (recrystallisation)	Temperature, residence time, choice of plasticisers, symbiotic polymers, storage conditions	Higher processing temperatures; reduce RT and storage time; optimize plasticiser content; chemical, enzymatic, or physical modification [131,132,133,134]
Moisture sensitivity	Hygroscopic nature, hydroxyl groups	Chemical modifications; hydrocolloid additives; increasing crystallinity [104,105]
Weak barrier properties	Hydrophilicity, low crystallinity, hydrogen bonding	Hydrophobic coatings; modify chains; use nanocomposites or nanofibrils; develop compatibilizers multilayer structures [132,133]

**Table 4:** Technical issues, causes and mitigation strategies in blended polymer films.

Technical Issues	Causes	Mitigation Strategies
Poor interfacial adhesion	Thermodynamic immiscibility, surface tension differences	Using compatibilizers (reactive, block copolymers); improving mixing procedure [140]
Agglomerates and heterogeneities	Insufficient mixing, polymer incompatibility, uneven compatibilizer distribution	Improving mixing procedure (e.g., use twin-screw extruders); melt blending techniques; optimizing compatibilizer concentration [140,141]
Loss of transparency	Difference in refractive indices, light scattering at phase interfaces	Select miscible polymers; enhancing mixing with compatibilizers [140,143]
Inconsistent mechanical properties	Phase separation, heterogeneity	Optimizing blend ratios; ensuring proper compatibilization, improving blending; refining processing conditions [141]
Uneven thickness	Differences in polymer rheology	Select polymers with compatible viscosities; using proper compatibilization strategies; adjust processing settings [144]
Processing instability	Low melt strength, large viscosity differences	Using high-melt-strength polymers; adding compatibilizers; optimizing processing parameters; using multilayer architectures [139,144]
Thermal degradation	Different degradation temperatures, narrow processing windows	Control of temperature, moisture, and blend composition; adding PPAs; optimizing processing conditions (defining optimal processing window) [139]

**Table 5:** Technical issues, causes, and mitigation strategies in multilayer films.

Technical Issues	Causes	Mitigation Strategies
Curling	Differences in crystallinity, thermal expansion coefficients, and residual stresses	Balance layer thickness; use compatible resins; add ionomers, increasing cooling rates; predictive modelling [146,147]
Interfacial instability	Mismatched viscosities and elasticities between layers	Select matching rheological polymers; control melt and die temperature; use well-designed feedblocks and dies [147,148]
Layer breakage	High shear stresses, thin layers	Adjust output rate and die geometry; use materials with higher melt strength [147,148]
Poor adhesion	Incompatible polymer chains	Use adhesive resins or tie layers; optimize processing conditions [22,122,147]
Non-uniform thickness	Uneven melt flow	Design better dies and melt flows; monitoring MFR and pressures [22,147]
Predicting properties difficult	Properties influenced by several parameters	Test final films under realistic conditions [146,147]
Recycling difficulties	Complex structure, chemical incompatibility	Adopt design-for-recycling principles [22,122]

**Table 6:** Technical issues, causes and mitigation strategies in MDO films.

Technical Issues	Causes	Mitigation Strategies
Stretch resonance and instability	Uneven stretching, lack of LCB	Use PPAs; select higher MW polymers (e.g., HMW-HDPE) [25]
Breakage	High stretching force, high material density	Optimize resin density and crystallinity; ensure even stretching conditions [27]
Localized thinning	High crystallinity, densely packed fibrils	Reduce crystallinity, prevent large crystalline spherulites, optimize density [25,27,150]
Orientation inconsistency	MDO stretch	Fine-tuning MDO stretch ratio to balance MD and TD strength [151]
Defects (e.g., point gels, fibre defects, haze) in recycled materials	NIAS (impurities and gel particles)	Reduce recycled material fraction, minimize crosslinking, higher LLDPE content, improve PCW quality, optimize processing conditions [22,25]

## Data Availability

The original data presented in the study are openly available from the cited sources.

## List of Abbreviations

AI	Artificial intelligence
BUR	Blow-up ratio
CAGR	Compound annual growth rate
EU	European Union
EVOH	Ethylene vinyl alcohol
FLH	Frost line height
HBP	Hyperbranched polymer
HDPE	High-density polyethylene
HMW-HDPE	High-molecular-weight high-density polyethylene
LCB	Long-chain branching
LDPE	Low-density polyethylene
LLDPE	Linear low-density polyethylene
MAH	Maleic anhydride
MD	Machine direction
MDO	Machine direction-oriented
MFR	Melt flow rate
MMW-HDPE	Medium-molecular-weight high-density polyethylene
MW	Molecular weight
MWD	Molecular weight distribution
NIAS	Non-intentionally added substances
NIR	Near-infrared
PA	Polyamide
PCW	Post-consumer waste
PCL	Polycaprolactone
PE	Polyethylene
PEG	Polyethylene glycol
PET	Polyethylene terephthalate
PGA	Polyglycolic acid
PHA	Polyhydroxyalkanoates
PHB	Polyhydroxybutyrate
PLA	Polylactic acid
PP	Polypropylene
PPA	Polymer processing additive
PVOH	Polyvinyl alcohol
PVT	Pressure-volume-temperature
RT	Residence time
SCB	Short-chain branching
TD	Transverse direction
TUR	Take-up ratio
UV	Ultraviolet
WVTR	Water vapour transmission rate
